# Early detection of human impacts using acoustic monitoring: An example with forest elephants

**DOI:** 10.1371/journal.pone.0306932

**Published:** 2024-07-26

**Authors:** Peter H. Wrege, Frelcia Bien-Dorvillon Bambi, Phael Jackel Ferdy Malonga, Onesi Jared Samba, Terry Brncic

**Affiliations:** 1 Cornell Lab of Ornithology, Ithaca, New York, United States of America; 2 Wildlife Conservation Society, Congo Program, Brazzaville, Republic of Congo; 3 Nouabalé-Ndoki Foundation, Brazzaville, Republic of Congo; 4 Zambian Carnivore Programme, Nkwali Camp, Mfuwe, Zambia; Bowling Green State University, UNITED STATES OF AMERICA

## Abstract

The impacts of human activities and climate change on animal populations often take considerable time before they are reflected in typical measures of population health such as population size, demography, and landscape use. Earlier detection of such impacts could enhance the effectiveness of conservation strategies, particularly for species with slow population growth. Passive acoustic monitoring is increasingly used to estimate occupancy and population size, but this tool can also monitor subtle shifts in behavior that might be early indicators of changing impacts. Here we use data from an acoustic grid, monitoring 1250 km^2^ of forest in northern Republic of Congo, to study how forest elephants (*Loxodonta cyclotis*) assess risk associated with human impacts across a landscape that includes a national park as well as active and inactive logging concessions. By quantifying emerging patterns of behavior at the population level, arising from individual-based decisions, we gain an understanding of how elephants perceive their landscape along an axis of human disturbance. Forest elephants in relatively undisturbed forests are active nearly equally day and night. However, they become more nocturnal when exposed to a perceived risk such as poaching. We assessed elephant perception of risk by monitoring changes in the likelihood of nocturnal vocal activity relative to differing levels of human activity. We show that logging is perceived to be a risk on moderate time and small spatial scales, but with little effect on elephant density. However, risk avoidance persisted in areas with relatively easy access to poachers and in more open habitats where poaching has historically been concentrated. Increased nocturnal activity is a common response in many animals to human intrusion on the landscape. Provided a species is acoustically active, passive acoustic monitoring can measure changes in human impact at early stages of such change, informing management priorities.

## Introduction

The maintenance of biodiversity is increasingly critical with climate change because diverse systems sustain the complex interactions that maintain the stability of ecosystems; ecosystems that have inherent value but also provide services of value to human society [1–4]. Monitoring biodiversity is a herculean task even when necessarily only a few species are monitored as indicators of overall diversity. Factors that negatively impact animal populations often take considerable time to be detected using metrics like population size, sex ratio, and recruitment, with the consequence that problems are recognized later than would be optimal. There is high value in detecting impacts at early stages, because conservation efforts would likely result in better outcomes and often at lower cost. We suggest that passive acoustic monitoring is one tool that could track such impacts.

Historically, conservation strategy has been based primarily on monitoring change in the population size and distribution of select species and mitigating the more obvious human impacts. Population monitoring is often time and effort intensive (e.g. transects) and difficult to scale to large landscapes and for species that range widely (e.g. camera traps, satellite collars). These constraints often result in long periods between repeated censusing efforts, and detection of steep population declines later than would be optimal for implementing plans to ameliorate loss.

A more nuanced understanding of landscape-level use by key species may be critical for truly effective conservation. Nearly continuous monitoring that could detect changes in use at small temporal scales could provide indicators of emerging problems that have a time lag before their effect on population size and/or distribution is measurable. Such information-rich data, particularly at the level of populations, is difficult to obtain. One of the more promising approaches has been use of satellite telemetry, especially given the huge improvement in lifetime of transmitters, their size and cost, and the cost of transmitting data. But this approach can suffer from typically rather small numbers of individuals monitored and small samples can increase the influence of individual-specific response and choice [5–9], both of which can limit inference to populations. Recently, passive acoustic monitoring (PAM) has emerged as a cost-effective approach to such monitoring, potentially revealing population-level decisions on movement, feeding ecology, reproduction, and landscape use [10–13]. As a tool, PAM stands out for its potential to investigate particularly cryptic species as well as those active both day and night, when other methods have limited coverage or different precision day versus night.

Here we show how PAM can reveal population-level outcomes of individual behavioral response to assessment of risk—their ‘landscape of fear’, in a population of forest elephants (*Loxodonta cyclotis*) in northern Congo. Adult forest elephants likely have no natural predators, but a landscape of fear—the concept that spatial heterogeneity in perceived risk of predation is projected onto the landscape by prey species, influencing their occupancy in space and time [e.g. 14, 15]—has been created due to poaching for ivory and increasing human population density throughout their range. Forest elephants respond to these risks in various ways, for example avoidance and range restrictions, and by shifting to increased nocturnal activity [16–19]. Changing diel pattern is a frequent response to risk in many mammals [20–22] and shifts in this pattern could be monitored as a population-level indicator of risk perception.

Using data from an ongoing PAM study in northern Republic of Congo, we focus on how forest elephants perceive the risk due to logging activities and the overall pattern of illegal hunting. With the study area divided into three strata with different levels of human activity, namely, protected national park, active logging concession and a logging concession closed for many years, the potential landscape of fear varied temporally as well as spatially. Our goal was to better understand how differing conditions affected elephants’ assessment of risk (indicated by variation in proportion of nocturnal activity as well as by spatial movement) and how they weighed the costs of response. Acoustic monitoring successfully revealed nuanced behavior shifts on fine spatial and temporal scales, broadly supporting *a priori* predictions. But this study also revealed diel behavior patterns not clearly associated with any known sources of risk, highlighting the key importance of obtaining complementary data through other monitoring approaches that excel in revealing different aspects of an organism’s ecology.

## Methods

### Forest elephant vocal behavior

Variation in the frequency of recorded elephant rumble calls is the basic datum used in PAM studies of forest elephants. It is therefore important to justify why we argue that counts of elephant rumbles can be used as a metric of activity (i.e. not resting/sleeping), movement in the landscape, and local density. The acoustic cue used in these studies is the very low frequency ‘rumble’ vocalization (dominant frequency 31 Hz; [23]), a call type that is used predominantly in affiliative social contexts and family group coordination [24] and is the most common call type emitted by forest elephants. Direct counts of individuals, combined with localization of calls in simultaneous recordings, show that the number of calls in a given time period correlate well with the number of individuals present [25]. Individual variation in calling rate is of little consequence because calls are aggregated over time, from multiple individuals. This method was used to estimate the population size of forest elephants in a national park in Ghana, the estimate comparing well with simultaneous estimates based on dung counts along transects and from genetic mark-recapture [26]. Estimating calling rate requires visual counts of the animals available to call and, for forest elephants, this has only been possible at forest clearings. Forest elephants aggregate at these clearings for access to minerals and for social interaction. Densities in clearings can be quite high compared to those typical within the forest. Although the acoustic-based estimate of population size compared well with other methods [26], we suspect that calling rates might often be lower when elephants are moving through the forest, because they travel in small family groups [27, 28] and the social environment is less rich. If true, this would change the slope of the relationship between call rate and density, but not the relationship that increased calls correlates with increased elephant numbers.

All PAM studies depend on ‘detection distance’—the distance between the recorder microphone and the source of the signal, which determines the landscape area sampled by a single recorder. This distance is affected by many factors, including microphone sensitivity to different frequencies, habitat structure, temperature and humidity, and masking sounds in the environment. Microphone sensitivity is primarily an issue when using or comparing different recording hardware. Detection distance was determined empirically for this study in mixed species forest (S2 Text), but attenuation of the low frequency signals used in this study are minimally affected by forest structure (several studies summarized by [29]) and therefore detection distance is not corrected for habitat type. Similarly, sound attenuation increases with humidity, but again attenuation of the low frequency signals used here is negligible (<0.1 db/km) for the full range of humidity found in these forests [30].

### Study site and acoustic grid

**Fig 1:**
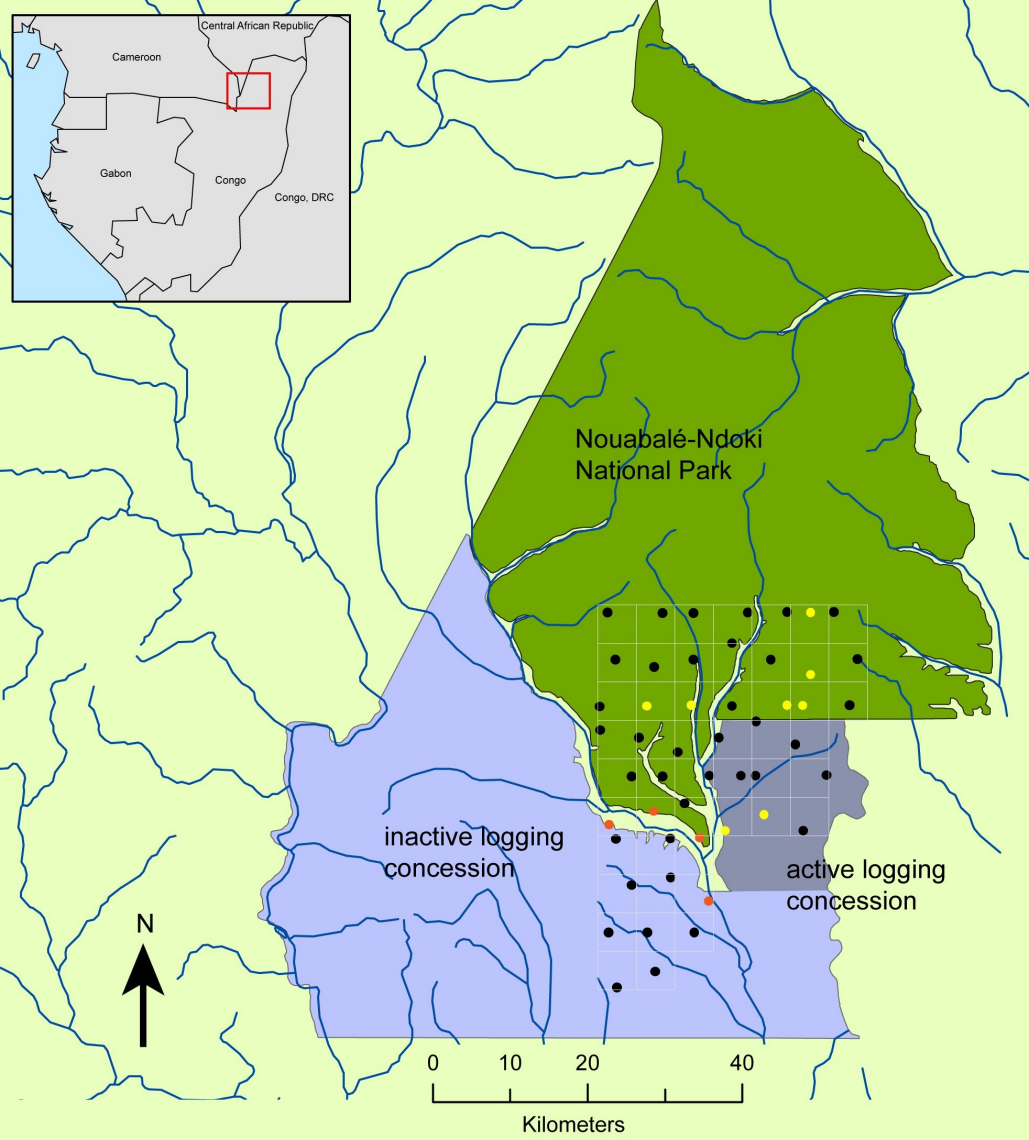
Study area in northern Republic of Congo. 50 acoustic recorders (forest type: black = mixed, yellow = monodominant, orange = open) in southern national park and adjacent logging concession (darker purple active logging, lighter purple inactive logging). Basemap files courtesy of ESRI. ESRI reserves the right to grant permission for any other use of the image.

This study is part of an ongoing passive acoustic monitoring project located in the southern part of Nouabalé-Ndoki National Park and parts of an adjacent forestry concession in the Guineo-Congolean biogeographical region of the Republic of Congo (Fig 1). This study includes data from the first 3.5 years of study, 15 December 2017 to 27 April 2021, at which time recorders were replaced with a system differing in sensitivity. We intended for all recording units to record continuously throughout this period, although some gaps occurred due to battery/equipment failure. Fifty acoustic recording units were installed in the study area across three strata that represent differences in human impacts: an integrally protected area (‘national park’, 29 sites, 725 km^2^ monitored), a sector of the logging concession actively logged from 2014–2019 (‘active logging’, 10 sites, 250 km^2^ monitored), and an inactive sector of the logging concession last exploited in 2006–2007 (‘inactive logging’, 11 sites, 275 km^2^ monitored). A 5km grid was superimposed on the 1250 km^2^ study region and systematic random placement used to site one acoustic recorder within each 25km^2^ grid cell (mean inter-recorder distance: 5.5 km, SD 1.4; S1 Text). Acoustic devices were designed and manufactured by the Cornell University K. Lisa Yang Center for Conservation Bioacoustics [31]. Recorders were scheduled to acquire acoustic data continuously, at a sampling rate of 8 kHz and 16-bit resolution. Batteries were replaced and acoustic data were retrieved from the recorders approximately every 4 months. Microphone sensitivity was empirically determined to successfully record an average forest elephant rumble vocalization at 540m (Fig a and b in S2 Text). Designation of forest type at each recording site reflected the predominant forest canopy within 600m of each recorder, based on an analysis of satellite images distinguishing mixed forest, monodominant forest, and open canopy [32]. The national park stratum, because of its protected status, was considered the ‘control’ stratum in these analyses and provided the best baseline expectation for elephants use and behavior against which to compare elephant behavior in the other two strata. The main vegetation types, which varied in their proportional representation across the three strata (Table 1), are *terra firma* mixed forest, monodominant *Gilbertiodendron dewevrai* forest, and predominantly open canopy (four sites in the floodplane of the Ndoki River). The forest structure of the inactive logging stratum was modified by previous selective logging, which created more canopy gaps and logging tracks, promoting growth of understory herbaceous species. This stratum also uniquely has many open areas, or *eyangas*, mostly to the west of the recording grid, which may be attractive to elephants. The Ndoki River flows between the three strata, and the slightly smaller Goualougo River separates part of the active logging stratum from the national park along the park’s western side (Fig 1).

**Table 1:** Predominant vegetation within 600m of each recording site, by stratum.

	forest type					
	mixed		monodominant		open	
stratum	N	% in stratum	N	% in stratum	N	% in stratum
inactive logging	9	82	0	0	2	18
national park	21	72	6	21	2	7
active logging	8	80	2	20	0	0

See S6 Table for site-specific cover.

### Data extraction/reduction

Elephant rumble vocalizations and gunshots were tagged within sound files using automated detection algorithms. The rumble detector used acoustic feature extraction on Fourier transformed sound files, exploiting the relatively stable power distribution across frequencies at millisecond time scales and the harmonic distribution of the fundamental frequency in rumble calls [33]. The gunshot algorithm implemented a template cross correlation method based on example gunshot sound clips recorded in the field [18]. The rumble detector output typically resulted in more than 246,000 detections in a 4-month deployment (of which only about 4% are elephant rumbles), so for each deployment we randomly selected three days per week and hand-verified these selected dates at every site. In total we verified detector output for 28,786 days, averaging 576 days per site (CV = 10%, due to temporary recording failures at individual sites). Forest elephants often travel 8-9km/day [9, 34, 35], and so total calls at a given site can vary considerably from day to day. We pooled call counts for each week at each site. Weekly total calls, and the number at night, could be used directly in analyses of diel calling behavior, using the ‘trials/event’ syntax for binomial regression. However, for the few analyses modeling total calls per week, we estimated a value standardized for a 3-day count if more or fewer days were examined for a given week (e.g. in the first seven months we verified all dates; occasionally fewer than three days were available at a given site because recording had stopped). Gunshots were relatively rare events (total n = 319), so detector output from all recording days at all sites were hand-verified and false positives removed. Recorded gunshots included rapid automatic weapons fire as well as single discharges, which could have been from automatic weapons or from shotguns. While single shots could be bushmeat hunting events rather than elephant poaching, we assume here that any gunshot would be interpreted by elephants as an indication of poaching risk. Gunshots at a particular site, occurring within 30 minutes of one another, were grouped as a gunshot event (n = 86). Although this is an arbitrary time block, it would likely group multiple gunshots associated with one hunting/poaching event, while longer separations would most likely represent attempts on different animals. For this study, the total events per year, within each of the strata, was used heuristically as a measure of poaching risk, but not quantified in models.

### Prediction covariates

**Fig 2:**
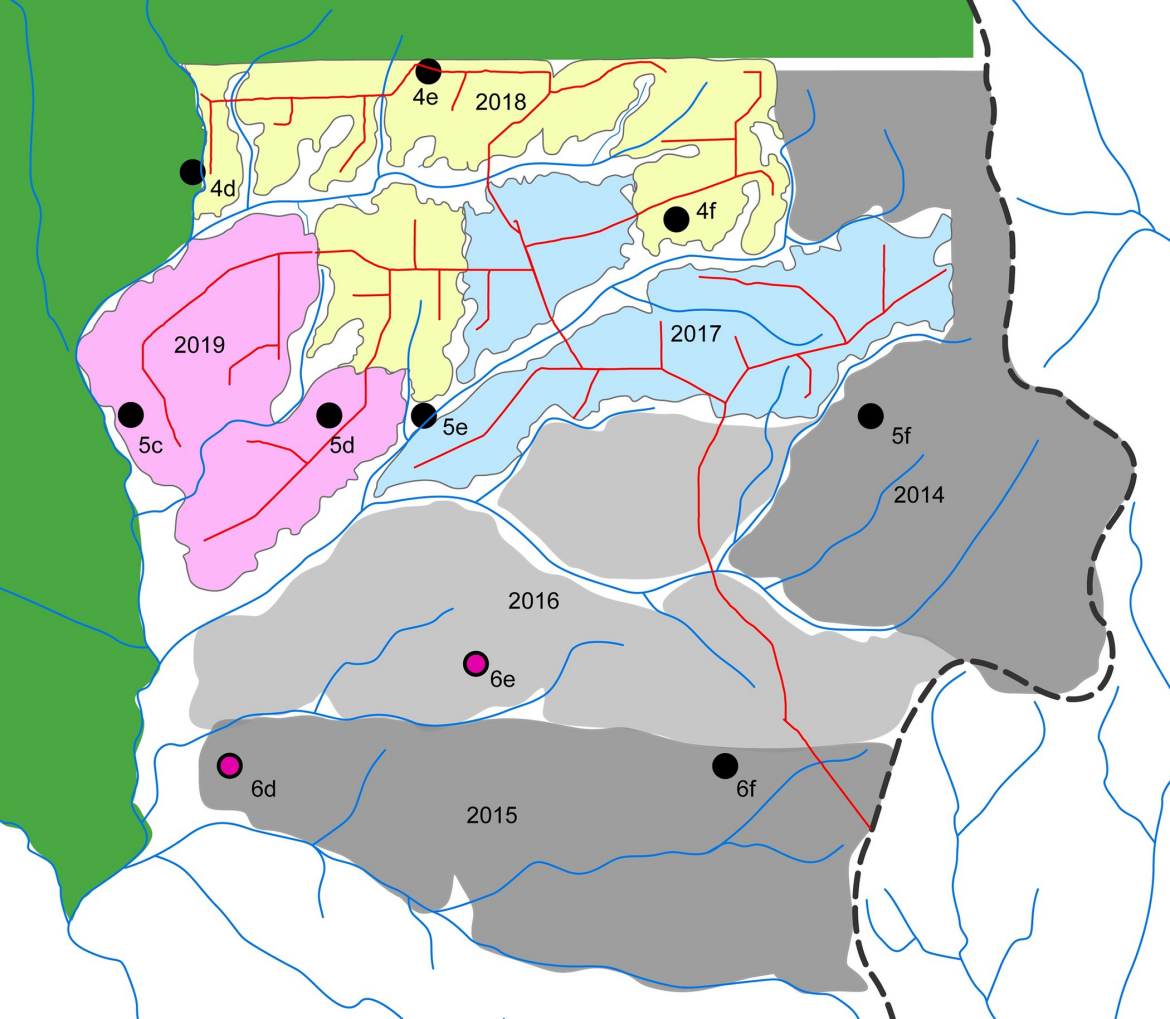
Zones of logging activity in the active logging stratum. Exploitation areas for each year were drawn as minimum convex polygons around the actual sites of felled trees. Logging roads (red), rivers (blue), acoustic recording sites dots (black = mixed forest site, magenta = monodominant forest site). Active logging was situated southeast of Nouabalé-Ndoki National Park (green area).

Patterns of elephant behavior in this forested environment were explored using a set of covariates at a fairly broad spatial and temporal scale, with primary analyses modeling the probability of nighttime calling activity. Daily rainfall data were available from Goualago Camp, a research site within the national park and roughly central in the acoustic grid. Months were considered dry season months if rainfall was less than 60mm [36]. Logging operations were ongoing within the active logging stratum from the beginning of the study in December 2017 through December 2019. As is typical of selective logging in central Africa, road construction and felling were densely concentrated in relatively small zones of the concession for multiple months (Fig 2 shaded areas). Tree-felling and extraction were concentrated in time and space even within these zones. Because there was no felling or extraction during December of any year (the start of our ‘study’ years), logging in 2018 corresponds to study year 1, while 2019 logging corresponds to study year 2. Logging intensity was much higher in year 2 than in year 1 (205 trees/km^2^ versus 125 trees/km^2^, respectively). Larger logging roads were active nearly continuously until logging in that zone was complete. The categorical covariate ‘logging exposure’ captured the temporal history of logging in each zone of the logging concession. All sites located anywhere within a zone exploited in a particular year (Fig 2) had the same covariate value. We created eight levels for the logging covariate, including the time periods before and during logging (pre-exposure, active), and categories for each year after logging was completed in a zone (done 1^st^year-done 6^th^year). Although the majority of impact due to logging would have occurred within the years indicated, logging roads were not always closed off, and some additional felling may have occurred after indicated dates.

Definitions of analysis covariates, the related predictions examined in our models, and an indication of support in our data, are listed in Table 2. The dearth of detailed data about forest elephant ecology and behavior means that there are few *a priori* hypotheses available to underpin the predictions in Table 2 [but see 17–19, 34, 35, 37]. We included a covariate measuring the total number of calls per time period (call density), a proxy for elephants density [25], based on the idea that either competition or ‘safety in numbers’ could affect the behavioral outcome of risk assessment.

**Table 2:** Covariates used to model elephant behavior in the Nouabalé-Ndoki landscape and possible *a priori* predictions for nocturnal activity.

Covariate	Description	Prediction re p(night calls)	Analysis Support (% nocturnal)
study year	categorical variable for study year, beginning 15 December.	no change	no–generally decreased through study
season	wet vs. dry (dry months <60mm cumulative rainfall).	no change	no–significant differences, direction dependent on stratum
stratum	national park active logging inactive logging	~equal proportions day/night. high in years1-2, decreased in remaining period ~equal proportions day/night.	yes yes/no–high but remained high no–intermediate between national park and active logging
forest	mixed—terra-firma forest, including sub-types with relatively open understory, dense understory, and prominent liana growth forms. monodominant—tall closed-canopy open–sparse canopy	~ equal day/night. ~ equal day/night higher than other habitats	~ yes no—variable yes
logging exposure	pre exposure–recorder within area subsequently logged active–recorder within area logged that year done 1^st^year-done 6^th^year–recorder within area where logging ended 1–6 years previous.	~equal day/night higher declining with time since active logging	no–highest levels (small sample) yes no—persistence of nocturnal bias for many years
call density	continuous covariate for total number of calls recorded per week/site (measures relative animal density)	low perception of risk: competition pushes more individuals into night activity high perception of risk: ‘safety in numbers’ and competition would decrease nocturnal activity	complex influence only partly consistent with prediction

### Statistical methods

Statistical analyses were performed in SAS^©^ 9.4 and JMP Pro^©^ 16.1. We used binomial models (logit link) to investigate diel calling behavior, with events/trials syntax. We modeled the likelihood of night calling, with the day period defined as 06:00–17:59 hrs. The model included covariates for temporal and ecological factors that previous studies had shown might affect forest elephant behavior, as well as two covariates, exposure to logging and call density (an index of elephant density; see Methods section one), that specifically might influence the likelihood of nocturnal behavior.

The binomial model was applied to the entire study system, but our focus was on application to each of the strata independently, particularly because the logging impacts were specific to only one of the three. We did not use any model selection approaches, choosing to control for influence of each of the covariates whether or not explaining significant variation, and including only two-way interactions in the interest of interpretation. We used ‘dummy’ coding for categorical variables, with the fourth study year, wet season, the national park stratum, and mixed forest as reference levels. Although the covariate for study year could be used as a continuous predictor, we used discrete, unordered, levels because we had no *a priori* prediction that relationships with nocturnal behavior would be linear across years. We modeled changes in call density (∞ elephant density) in response to logging with a zero-inflated negative binomial model.

Statistical significance was taken to be satisfied if the probability of Type I error was less than 5 percent. Tests among class-level adjusted means (least squared means) used Scheffe adjustments for multiple comparisons. Note that estimates of the LSM for a given factor hold other model covariates at their mean levels, so absolute values for probability of night calling are less informative than the relative values across classes. In discussing patterns of diel behavior, we refer to the probability or change in probability of nocturnal calling by back-calculating from the model odds ratio.

## Results

**Fig 3:**
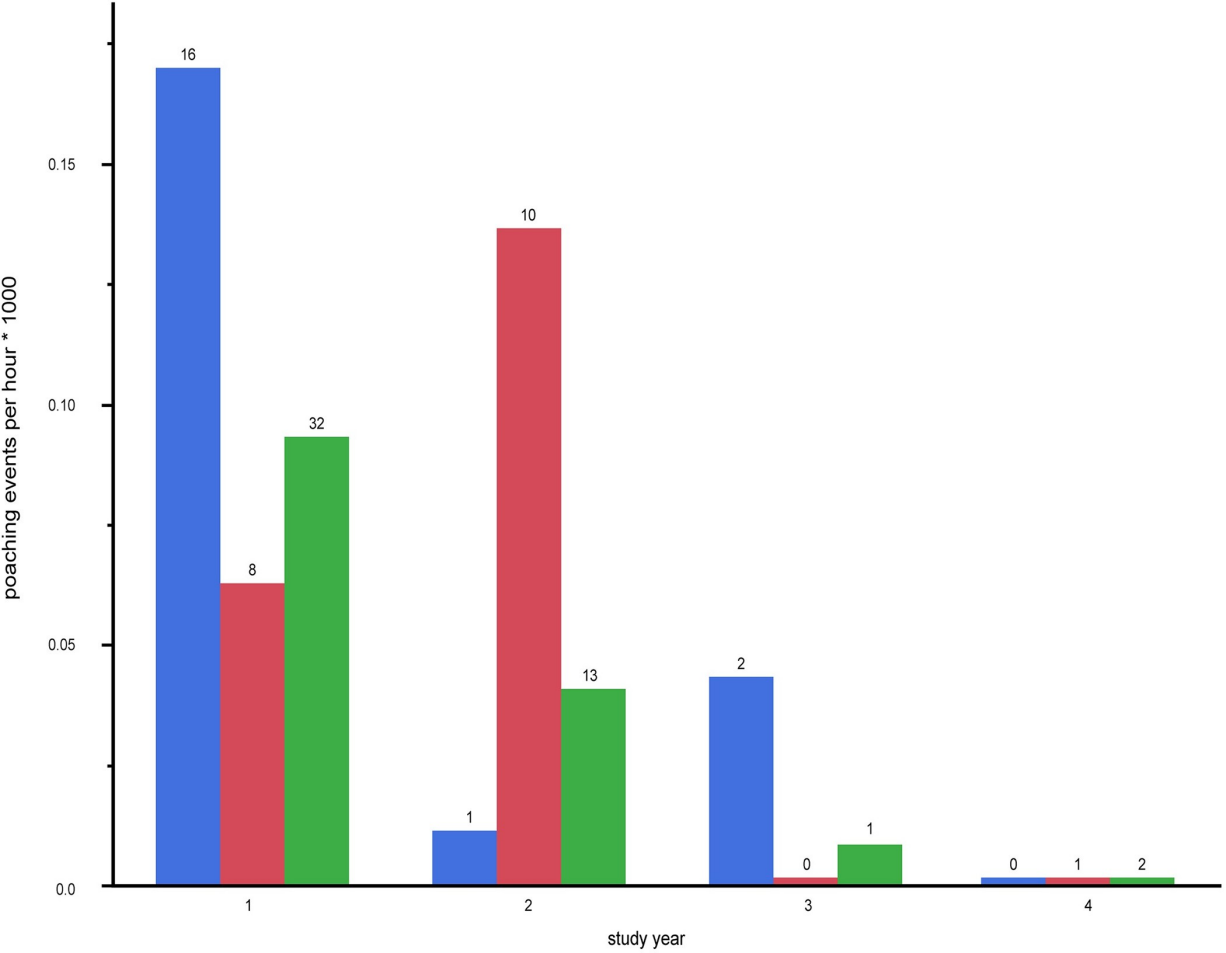
Acoustically detected gunshot events by stratum. Bar heights are standardized for total hours of recording in each stratum/year. Actual number of shot events over each bar. inactive logging—blue, active logging—red, national park—green.

Poaching pressure is arguably the human activity that most contributes to a forest elephant’s landscape of fear. Although we recorded 322 gunshots over the 3.5 years of study, 98 percent of these occurred in the first two years and were distributed over many different months. These were distributed too sparsely over time to be directly included in our models of nocturnal calling. For this reason, we use the pattern of gunshots in space and time heuristically, to further inform our interpretation of the determinants of nocturnal behavior in the study population of elephants. Fig 3 shows that the frequency of recorded gunshot events decreased strongly through the study period (correlation with year, across strata: r = -0.70, p = 0.02). This precipitous decline was due to high investment by the Fondation Nouabalé-Ndoki in professionalizing the anti-poaching program, beginning in study year 1.

**Fig 4:**
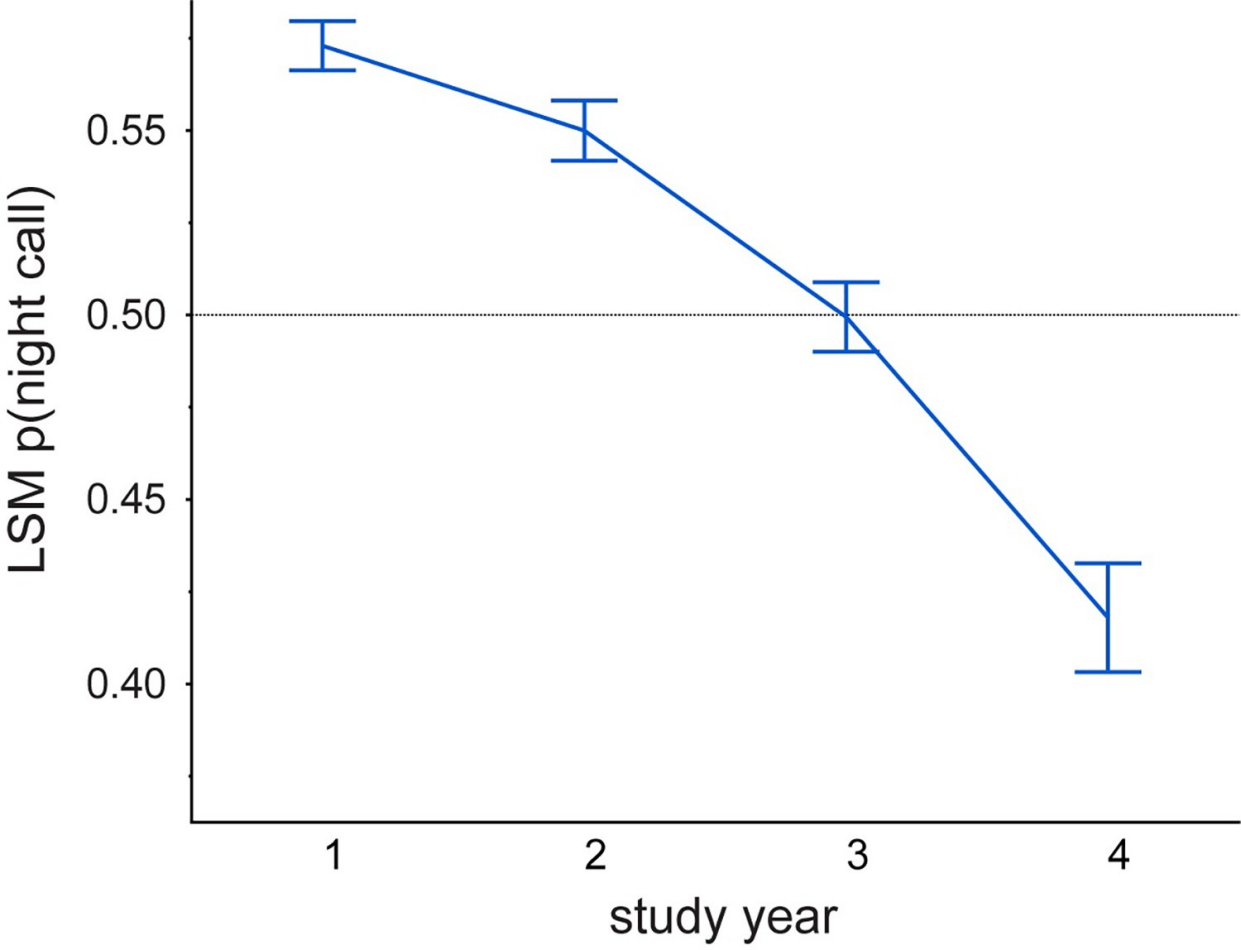
Change over time in proportion nocturnal activity across the study system. All comparisons between years significant at p<0.001 except year one and two (ns). Error bars are SE of the LSM.

In this study the overall probability of a call being recorded at night was 0.491 and the basic model, applied to the entire study area, showed most predictors highly significantly affecting the likelihood of nocturnal calling behavior (Table 3). There was a significant and monotonic decrease in nocturnal activity through the 3.5 years (Fig 4). As predicted, nocturnal activity was significantly higher, overall, in the active logging area followed by inactive logging, with the national park having the lowest level of nocturnal activity (LSM (SE): active 0.55 (0.01), inactive 0.53 (0.008), park 0.45 (0.007); all pairwise comparisons significant).

**Table 3:** Likelihood ratio statistics for the binomial model applied to the whole study system and separately for each stratum.

		Entire Study Area (n = 48301 rumbles)		national park (n = 23686 rumbles)		inactive logging (n = 19029 rumbles)		active logging (n = 5586 rumbles)	
Source	DF	Chi-Square	P value	Chi-Square	P value	Chi-Square	P value	Chi-Square	P value
study year	3	135.37	<0.001	72.84	<0.001	105.85	<0.001	10.92	0.0121
stratum	2	114.51	<0.001	na	na	na	na	na	na
season	1	0.67	0.414	102.58	<0.001	34.99	<0.001	14.00	0.0002
call density	1	35.05	<0.001	91.88	<0.001	408.99	< .0001	3.14	0.0766
forest	2^a^	1685.60	<0.001	381.30	<0.001	749.57	< .0001	16.70	< .0001
study year*stratum	6	145.93	<0.001	na	na	na	na	na	na
study year*season	3	19.16	<0.001	54.97	<0.001	31.32	<0.001	9.99	0.0187
study year*forest	6^a^	58.12	<0.001	125.57	<0.001	196.53	<0.001	9.55	0.0228
season*forest	2^a^	22.65	<0.001	73.51	<0.001	14.99	<0.001	5.55	0.0185
call density*season	1	66.24	<0.001	194.60	<0.001	2.21	0.137	16.03	<0.001
call density*forest	2^a^	56.21	<0.001	48.10	<0.001	152.98	<0.001	3.87	0.0492
logging	7							184.04	< .0001
season*logging	7							127.52	< .0001
call density*logging	7							154.94	< .0001

Model for active logging includes the extra term for exposure to logging. The total number of rumbles (= trials) is shown for each stratum. (see S1–S4 Tables for details of parameter and variance estimates for these models).

^a^active logging and inactive logging strata each had only two forest types, while the national park had all three. DF for inactive logging and active logging are consequently half the values listed in the table for parameters including forest type.

To explore the role that human activity might play in this variation along both spatial and temporal axes, we examined diel behavior separately in the three strata comprising our study area (see Fig 1). Table 3 summarizes model results in each stratum. These strata differ primarily on exposure to logging activities, with the active logging stratum exposed to activities for two years of the study while the inactive logging area was logged more than a decade previous. Because the national park was the stratum most protected and buffered from human activities (roads, villages, logging, but not poaching), we considered the diel patterns in this area to be our baseline for comparison.

**Fig 5:**
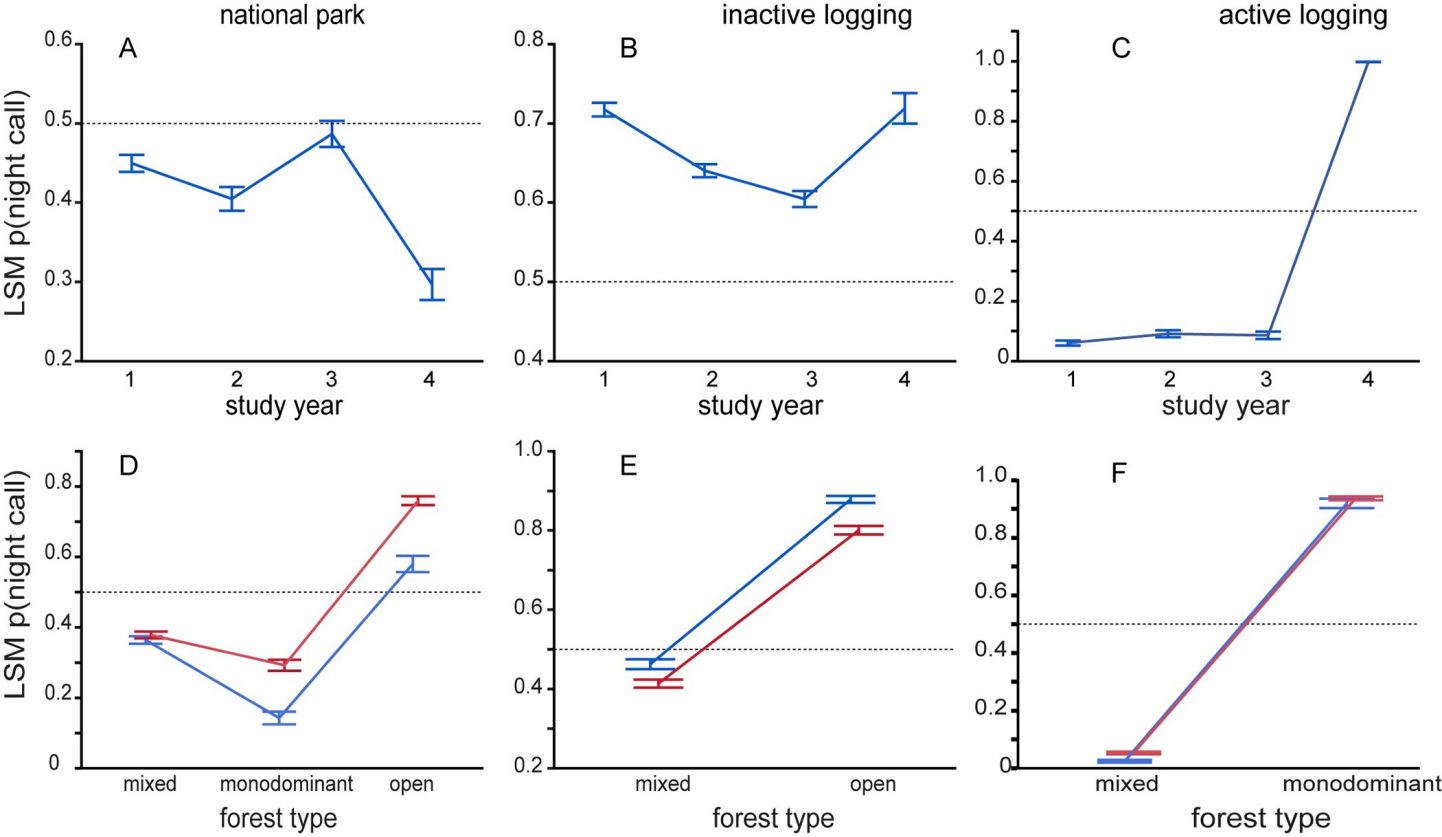
Plots of marginal means from binomial models applied to data from each stratum. Error bars are standard errors. Note that ordinant axes differ in scale, but all have a reference line at 50% probability of a call being at night. For all strata the plotted relationships were significant predictors of nocturnal calling. Interaction plots of forest type vs season: blue = dry season, red = wet season.

For the national park stratum, all covariates were highly significant (Table 3). Night calling trended lower over the study, although year three was higher than any other year (Fig 5A; between year comparisons all p<0.01 except 1vs2 p = 0.062 and 1vs3 p = ns). Nocturnal activity was significantly higher in the wet season compared to the dry season in monodominant and open forest, but not in mixed forest (Fig 5D). In either season, forest types differed significantly from one another (all p-values <0.001). Nocturnal activity predominated in open sites.

Elephants using the inactive logging stratum also trended toward less nocturnal activity through the study period but in study year 4 increased again dramatically (Fig 5B). In contrast to the national park stratum, inactive logging elephants were significantly less nocturnal in the wet season compared to the dry in both open and mixed forest sites (Fig 5E; p<0.001). Elephant activity was strongly nocturnal in open sites (Fig 5E; p<0.001 both seasons).

**Fig 6:**
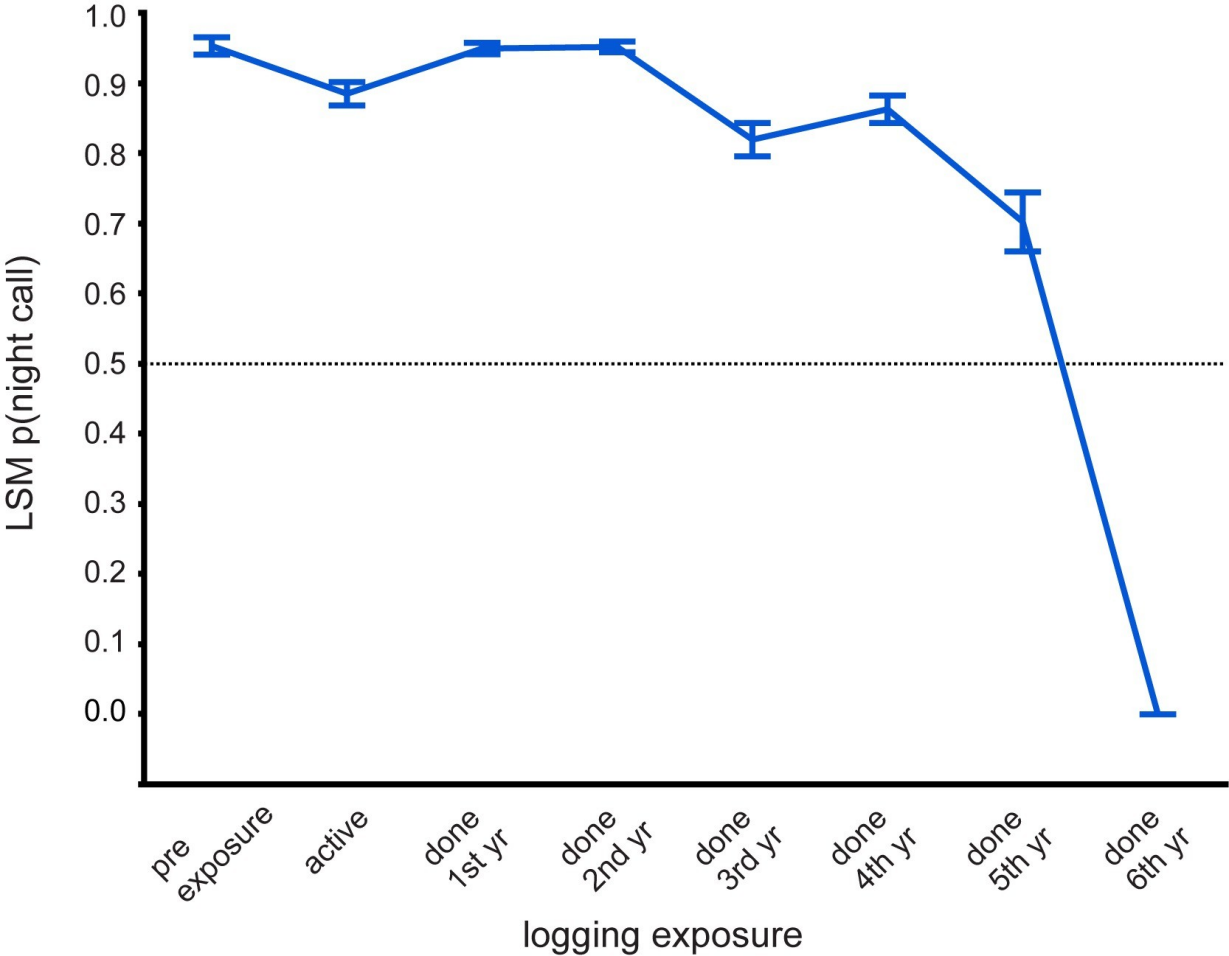
Behavioral response to exposure to logging activities in the active logging stratum. Error bars are SE. Most contrasts significant at p<0.05 (ns contrasts: pre-exposure vs done 1^st^year, done 2^nd^year; active vs done 2^nd^year, done 4^th^year; done 3^rd^year–done 5^th^year).

In the active logging stratum, most covariates were significant in the model, with those including logging exposure contributing most to explained variation (Table 3). Nocturnal activity remained relatively consistent for the first three years of study (year 1vs3 and year 2 vs3 not significantly different) but in year 4 nocturnal activity increased significantly relative to earlier years (Fig 5C), as in the inactive logging stratum. Forest type significantly affected the likelihood of night activity, but unlike in the national park, activity was more nocturnal in monodominant than in mixed forest in both seasons (Fig 5F). Level of exposure to logging activity, and its interaction with season and call density, had by far the strongest influence on night calling (Table 3). Fig 6 shows that forest elephant perception of risk from logging activities declined only gradually for over five years from when actual tree-felling stopped. The precipitous drop in the 6^th^ year after logging could be partially a sample size issue, as only 10 data points were available for estimating this level. Nocturnal behavior was unexpectedly high at the ‘pre exposure’ sites, which were predicted to be close to baseline and day biased.

**Fig 7:**
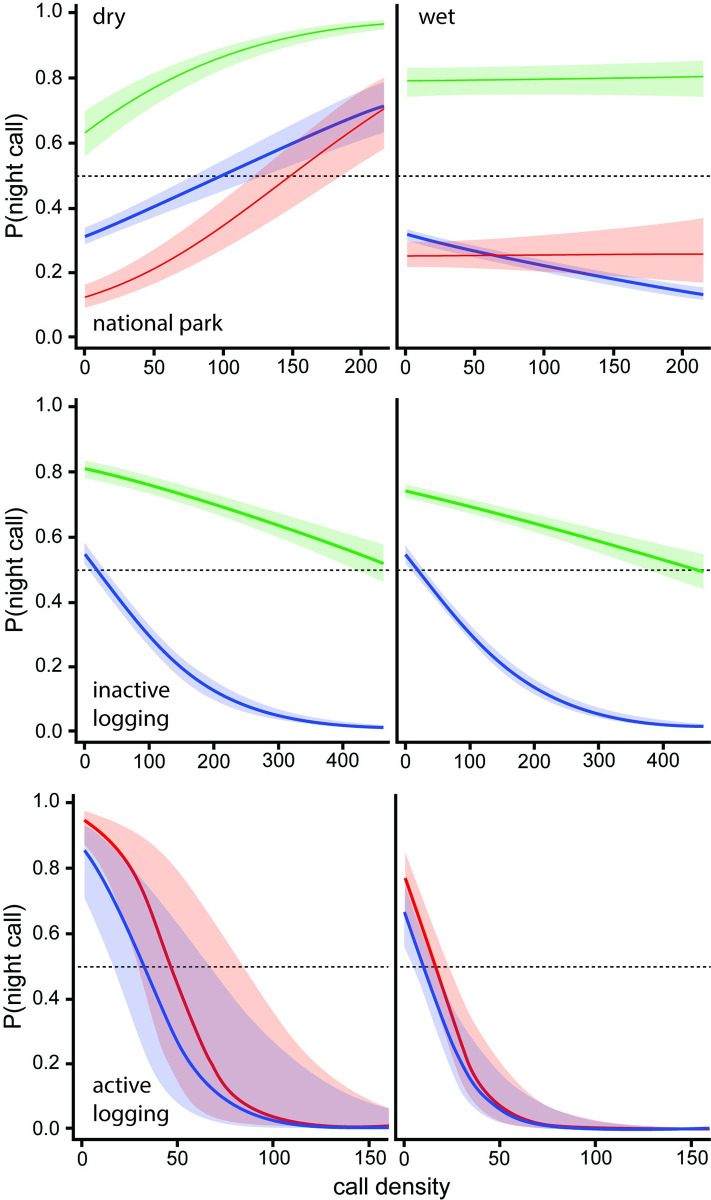
Call density effects on nocturnal behavior. Shaded areas are 95% confidence intervals. Blue = mixed forest, green = monodominant forest, red = open forest. Computed at study year = 2.

Call density was included in diel models as a proxy for animal density (see Methods), because increased density is expected to increase competition, which might affect the costs of changing activity pattern. During the dry season in the national park, night calling increased with density in all forest types (Fig 7). For monodominant and mixed forest, the relationship is consistent with the prediction that competition would push some individuals to less preferred nocturnal activity. But because nocturnal activity is preferred in open sites (Fig 5), we predicted that competition would increase daytime activity, which was not supported. During the wet season, mixed forest sites in the national park showed a decrease in night activity with increasing density, again contra prediction, while open and monodominant sites showed little change with density. In both of the logging strata, density affected nocturnal activity similarly across seasons, and was mostly consistent with prediction. In both strata increased density resulted in a shift away from the more preferred nocturnal time period (Fig 7). It is clear that these relationships are complex and not well explained by our *a priori* predictions. Particularly interesting is the steepness of change in the active logging stratum where only small increases in density generate a strong shift away from nocturnal activity.

**Fig 8:**
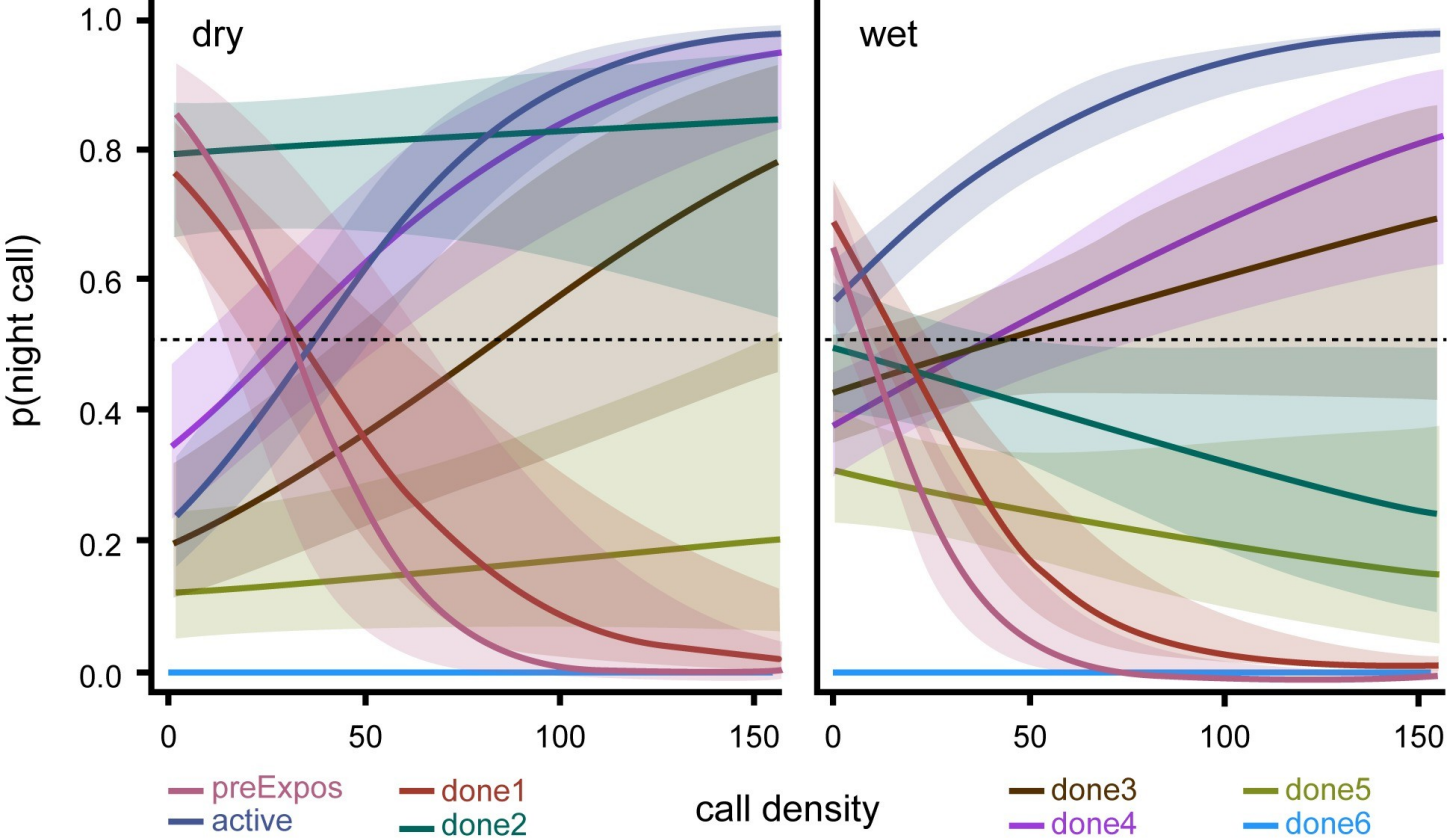
Predicted probability of night calls as call density changes in active logging, sliced by levels of logging exposure. Computed at study year = 2, forest type = mixed, with 95% confidence intervals indicated by shading.

The complexity of the density effect is particularly striking in the active logging stratum when estimates are sliced by levels of logging exposure (Fig 8). Although confidence intervals are often quite wide for some exposure classes, clearly there is quite a varied influence of density on elephant behavior. Our *a priori* predictions are too simplistic to account for this variability. A much more nuanced understanding of density effects seems necessary.

**Fig 9:**
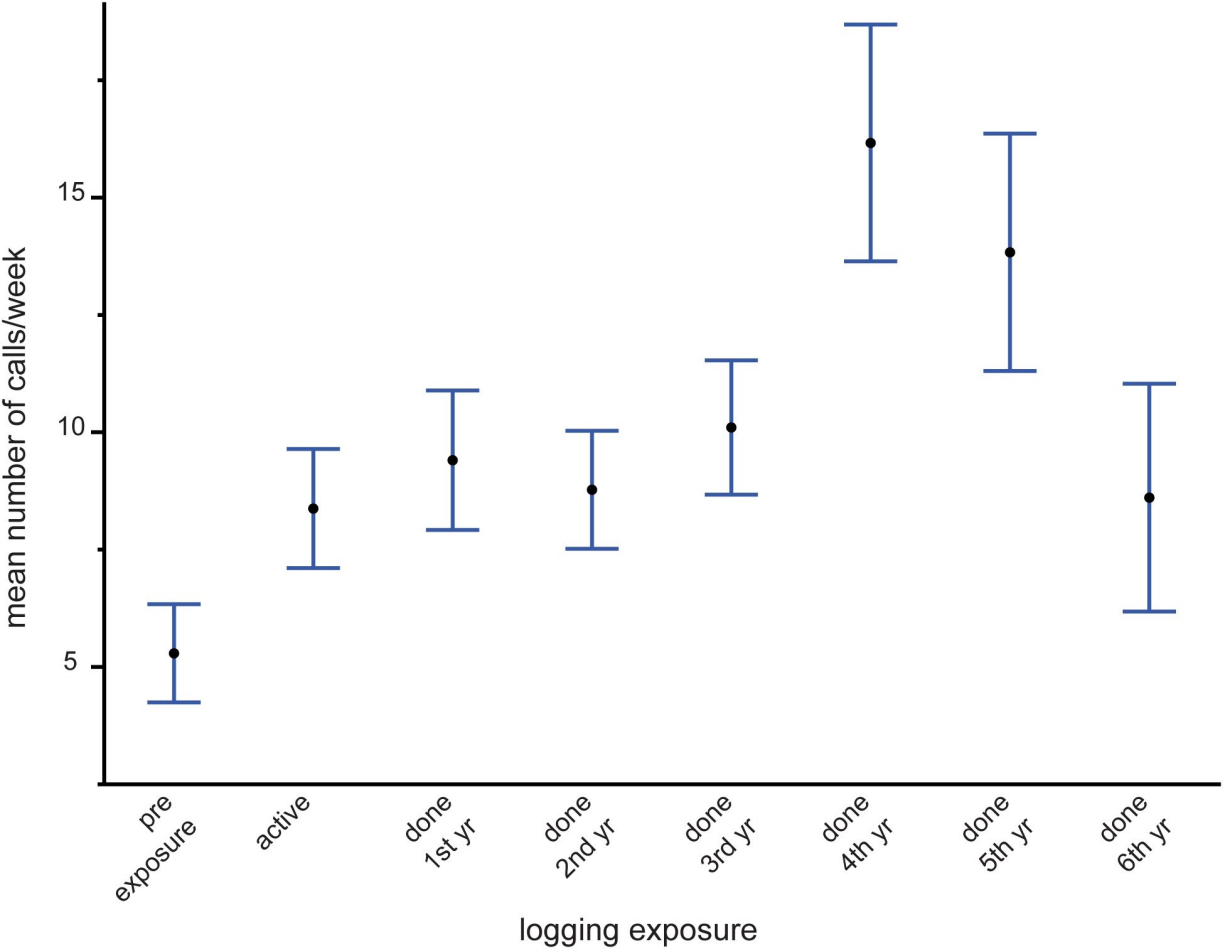
Change in numbers of calls near recording sites versus exposure to logging activity. Error bars are SE. Pre-exposure vs done 4^th^ yr, p = 0.04; active vs done 4^th^ yr, p<0.001; all other contrasts ns.

**Fig 10:**
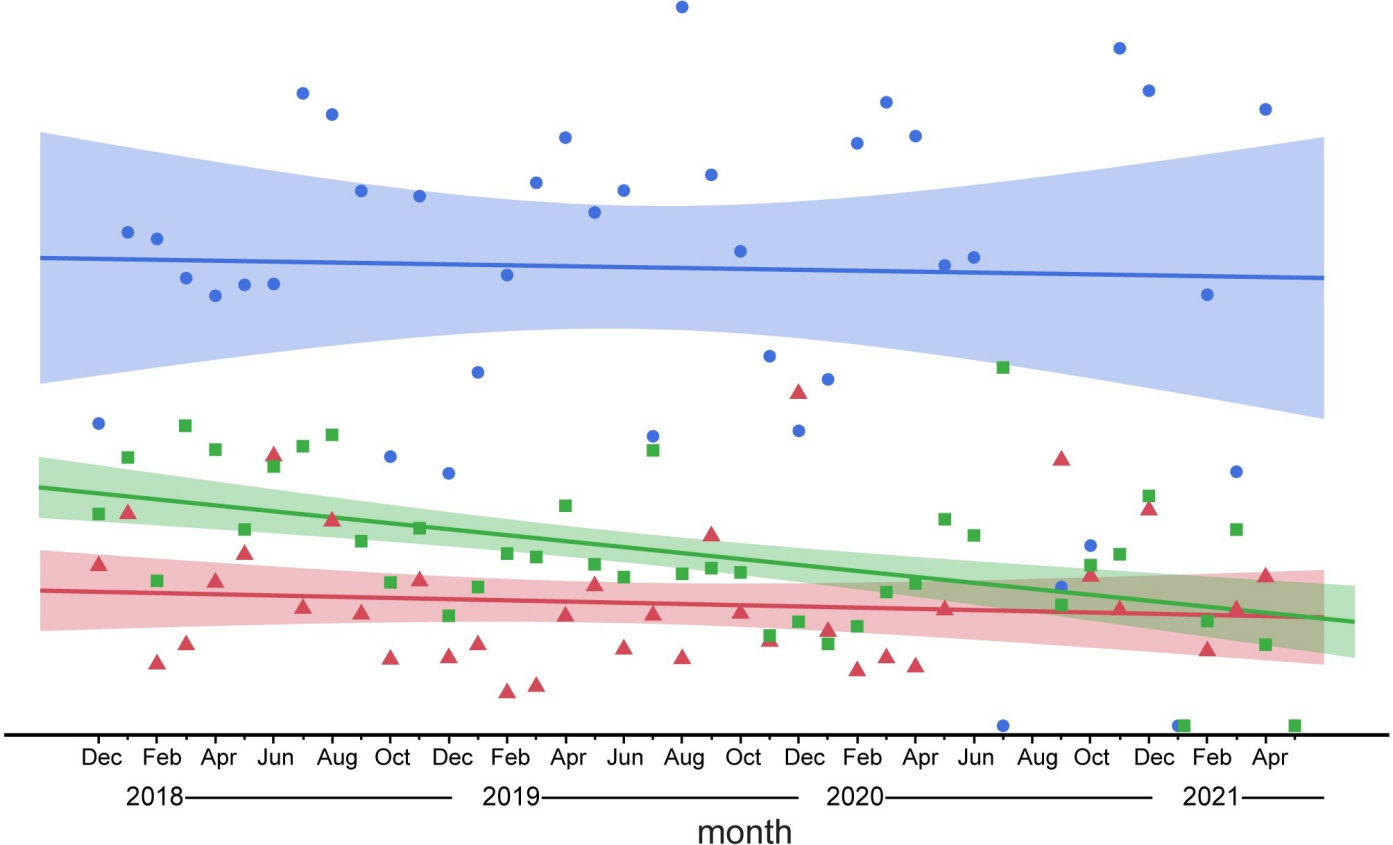
Relative call frequency in the three strata over 3.5 years of study. Calls per week are standardized (see methods). blue = inactive logging, green = national park, red = active logging.

Another possible behavioral response to perceived risk would be spatial movement out of higher risk areas. We examined this possibility by modeling total weekly calling activity (call density) along the axis of exposure using a zero-inflated binomial model (see S5 Table for details). We found minimal support for such a response in the active logging stratum based on a model including study year, season, forest type and logging exposure. Recalling that call density is correlated with number of elephants [25], Fig 9 suggests that the number of elephants did not change significantly until four or five years after logging activity in the area. Analyzed more broadly across the entire study system (Fig 10), numbers of elephants using either of the strata outside of the national park remained flat over the four years of study (in spite of decreasing perceived risk), while numbers appear to decrease inside the national park.

## Discussion

Passive acoustic monitoring, used to explore how an animal might perceive its environment and consequently modify behavior, further enhances the value of PAM for developing and implementing conservation strategies. While historical monitoring techniques can be used to model density and movement associated with habitat and seasonality, these would generally miss purely behavioral responses of target species to changed features of the landscape. Such behavior shifts might carry fitness costs and, in some cases, could anticipate both spatial movements within populations and population decline.

Forest elephants, even in the absence of any perception of risk, are active throughout a 24hr cycle. This may be necessary because of the time required to ingest sufficient nutrition for an animal of their mass. Based on acoustic studies in several different forest ecosystems [18, 19], elephants are slightly more diurnal than nocturnal when behavior patterns are viewed broadly. However, this study shows significant variation in nocturnality along several ecological axes (season, forest type, competition) for which we have no explanation. We found significant differences in nocturnal activity with season but have no hypothesis as to why this would be the case. Similarly, we found significant differences in the likelihood of nocturnal activity between mixed forest and monodominant forest areas. By contrast, open forest sites were expected to show much higher nocturnal activity because this pattern is seen throughout central Africa when elephants visit forest clearings. These unexplained patterns may or may not be related to any assessment of risk by the elephants and highlights the need for complementary monitoring methods that could explicitly tackle this variation.

When viewed across the entire study system, forest elephants reduced nocturnal activity within the diel period over the 3.5 years of study (Fig 4), which is consistent with the overall decrease in hunting activity during the same period (Fig 3). This overall trend appears to be driven mostly by the large proportion of sites in the most protected area, the national park, and in the inactive logging concession (Fig 5A and 5B). The active logging concession showed little change in the probability of nocturnal behavior through the first three full years of study (Fig 5C). Behavioral response to perceived risk was most clearly supported in this study by shifts in forest elephant diel activity in both the active logging concession and in open forest sites. Overall nocturnal activity was higher in the active concession than anywhere else in the study. The disruption due to logging, and possibly human activities after areas were formally closed, contributed to elephant’s ‘landscape of fear’ for a surprising number of years (Fig 6). Initially unexpected, nocturnal activity was highest where logging had not yet started (‘pre exposure’, Fig 6). These data, however, came from only two sites and only from one year. Significantly, one of these sites also recorded among the highest number of gunshots anywhere during the study, all during the dry season at the beginning of study year1. This observation, then, also supports the prediction that elephants will shift to increased nocturnal behavior when perceived risk is high. Except in the active logging area, we observed a trend of decreased nocturnal activity through the study period (Figs 4 and 5). We suggest that this was due to lowered risk assessment as poaching activity decreased dramatically across the study area. However, in the logging concession strata, both of which are more accessible to poachers than the national park, nocturnal activity jumped to higher levels in year 4 (Fig 5C and 5E). Although only data from December-April were available for this study year, the jump in night activity warns of a change in risk perception and highlights the potential value of this sort of monitoring.

Many studies have shown a nocturnal bias when forest elephants visit mineral clearings, or bais [e.g. 18, 38, 39]. This behavioral response to risk assessment was clearly supported by the very high nocturnal activity at open forest sites in this study, in both wet and dry seasons (Fig 5B and 5d). All four of the open sites in this study were along the floodplain of the Ndoki River, and the strongest predictor of poaching activity in this study system was proximity to large river systems [40].

We found only weak evidence that forest elephants actually abandoned areas where perceived risk was higher (indexed by call density; Figs 9 and 10). Although we estimated that there were higher numbers of elephants near recording sites that were more than three years post logging (Fig 9), this increase was not significantly different from the numbers of elephants in high exposure areas, and the trend was actually for lower numbers at sites that had been free of logging for the longest time. Fig 10 also shows little change in the density of elephants using either of the logging strata over the study period. In the active logging concession, the overall lowest numbers were at the two ‘pre exposure’ sites where our direct measure of poaching risk (recorded gunshots) was highest. This is consistent with a spatial shift by individual elephants at least from this highly impacted area. However, behavioral shifts in diurnal activity pattern (rather than spatial avoidance) might be a common strategy by forest elephants to reduce perceived risk. Elephants in Gabon exposed to transect-cutting and dynamite blasting for oil exploration became more nocturnal but did not leave the area of disturbance [19, 41].

Nocturnal activity in the inactive logging stratum fell between the higher levels in active logging and the lower levels in the national park. This elevated level of nocturnal activity in spite of logging activity having ended more than a decade previous. This stratum, as a formerly logged concession area, has considerable secondary vegetative growth that is known to be preferred by forest elephants [42]. This attractiveness is shown in the persistently high numbers of elephants using this stratum throughout the study (Fig 10). Apparently, elephants are accepting whatever costs come with shifting to more nocturnal activity for the benefits of better forage. What could be contributing to an apparent perception of higher risk?

Beirne et al. [9], using data from individual satellite tags, found interesting parallels in response to anthropogenic disturbance. On an annual time scale they found that individuals increased their nocturnal activity in response to disturbance, but were more diurnal at the monthly time scale [9]. They suggested that this might reflect consistent exposure (shift to night activity) versus transient exposure (no shift), very much like what might be the case in inactive logging versus active logging in this study.

Satellite canopy analyses classified more than twice as many pixels as ‘open’ in the inactive logging stratum compared to the average in the other two strata [32], indicating a generally more open canopy. Gunshot activity was particularly high in this area in the first year of study (Fig 3) but fell to quite low levels in succeeding years. Our analysis of active logging suggests that the perception of risk associated with logging persists for at least five years, but potentially declines after that. We feel the most parsimonious explanation for persistently high nocturnal activity is that the inactive logging area continues to be perceived by elephants to have higher poaching risk. This stratum is the most human-accessible of any in our study and included a hunting concession until shortly before our study began. For decades, access to this area by poachers has been relatively easy because of a large road to the south and the permanently navigable Ndoki River to the north. If correct, this interpretation in combination with the results from the active logging stratum, suggests that logging activity *per se* does not have a lasting effect on risk perception by elephants, but the subsequent increased accessibility to poachers does. This interpretation will be testable with the ongoing PAM monitoring in the now closed active logging concession area. In contrast to the post logging period in the inactive logging stratum, anti-poaching efforts now appear to be keeping poaching activity at very low levels throughout the study system, and so we predict increasing use of the active logging stratum as herbaceous vegetation increases, but for activity to be relatively equally distributed through 24 hours.

Finally, it is clear from Figs 7 and 8 that changes in the density of individuals has a strong, complex, but poorly understood influence on the distribution of activity over the diel period. Revealing this relationship shows one of the strengths of PAM, as it would be difficult to expose this interesting effect using other monitoring methods. In some cases, shifts in diel activity with density could indeed reflect competition or some interaction between competition and current risk assessment, but might also interact with food availability, forest type and seasonal movements. An initial assessment of whether competition is an important factor would be to quantify the frequency of ‘roar’ vocalizations as they relate to density. Hedwig et al. [24] showed that single roar vocalizations by forest elephants are almost exclusively given in agonistic interactions, especially over access to resources. These data would be available within the existing PAM sound files used in this study.

Carter and colleagues [43] suggest that temporal shifts by wildlife to reduce exposure to human activities, rather than abandonment, can be a mechanism for coexistence in an increasingly human dominated landscape. But this strategy probably does not come without fitness costs. Creel [44] reviews considerable evidence that proactive response to predation risk often incurs a fitness cost, likely associated with decreased foraging efficiency. Although olfactory cues are used extensively by elephants when foraging, shifting the feeding time-budget to more nocturnal foraging could result in decreased foraging efficiency [20]. Creel [44] predicts that proactive response to assessed risk will involve quite nuanced balancing against the costs, which might contribute to our observed complex relationship between density and nocturnal behavior (Figs 7 and 8), as well as the consistently high use of the inactive logging area in spite of perceived risk (Fig 10). Complementary study using other monitoring tools would help in teasing apart this complexity. For example, satellite telemetry would help to understand how long and how frequently individuals choose to be in portions of their landscape that they perceive as high risk. Given the mobility of elephants, it could be that individuals manage costs by regularly moving into and out of areas where forage potential is high, but also of high predation risk. Similarly, telemetry might illuminate the reasons that nocturnal activity varies with season and with mixed versus monodominant forest, by measuring movement speeds and the timing of directed movement over long distances. An achievable aspiration would be a catalogue mapping acoustic communication elements to behavior (e.g. indicating agonistic interaction, mating events, offspring alarm). With such a catalogue, PAM could simultaneously obtain data on population size and occupancy, and develop a landscape of competition intensity, mating intensity, and aspects of age structure.

## Conclusions

In this paper our focus is on risk assessment by a population of forest elephants and how this can inform us about how elephants perceive their landscape along an axis of human disturbance. The intent is to show how passive acoustic monitoring can be used to elucidate behaviors that are an early warning of negative impacts impinging on an animal population. We used PAM to show that forest elephants respond behaviorally to an assessment of risk from human disturbance, that this assessment can have both short and long temporal components, and that monitoring animal density would likely not detect this response. In a meta-analysis, Gaynor et. al. [21] show that shifts to increased nocturnality is a widespread mammalian response to human disturbance. If a species is ‘noisy’, PAM could be a widely applicable tool to assess human impacts at early stages.

Using PAM we are monitoring an emergent property, at the level of a population, rooted in the choices made by individual elephants. Individual-based data, for example from individual tagging systems, provide extremely valuable detailed information, but also are influenced by sometimes high variation between individuals [e.g. 9, 37], often small sample sizes, and, in the case of elephants, ethical issues with tranquilizing and fitting permanent telemetry collars. At the same time, this study also highlights the need to integrate other monitoring tools to truly improve the conservation prospects of such a complex and cryptic species as the forest elephant [e.g. 45].

By necessity, conservation often begins with the recognition of need but with only a crude understanding of the ecology and behavior of target organisms on the landscape. But truly effective strategies, with long-term positive outcomes, require an increasingly sophisticated understanding of organisms which, in turn, often requires a suite of monitoring tools that target various aspects of their ecology. Usually there will not be a ‘best’ tool; an increasingly nuanced understanding of conservation needs comes from application of multiple approaches which, logistically, may benefit through collaboration between expert practitioners. Passive acoustic monitoring is only one of these tools, although one with high value for continuous landscape-scale monitoring and with the potential to reveal emergent patterns of movement and behavior by animal populations.

## Supporting information

S1 Text PAM grid layout method. (PDF)

S2 Text Swift acoustic recorder detection distance for rumble calls. (PDF)

S1 Table Parameter estimates for entire study system. Binomial model.
(PDF)

S2 Table Parameter estimates for the national park stratum. Basic binomial model.
(PDF)

S3 Table Parameter estimates for the inactive logging stratum. Basic binomial model.
(PDF)

S4 Table Parameter estimates for the active logging stratum. Modified binomial model.
(PDF)

S5 Table Parameter estimates for the active logging stratum. Zero-inflated binomial model of call density.
(PDF)

S6 Table Forest type determination based on proportion of pixels in each of three classes. (PDF)
