# Supplementary material for: Early detection of human impacts using acoustic monitoring: An example with forest elephants
Source: PLoS One. 2024 Jul 26;19(7):e0306932. doi: 10.1371/journal.pone.0306932 (PMC11280225; doi:10.1371/journal.pone.0306932)
Supplement: S1 Text — (PDF) [file pone.0306932.s001.pdf]

S1 Text. PAM grid layout method. A random starting location was assigned; each site in that row was then assigned the same latitude but randomly shifted in longitude within the grid cell. The next grid row north or south of this starting row used the last random longitude position relative to the east boundary of the cell as fixed for the row, and the latitudinal position shifted randomly within the grid cell. Thus, random latitude and longitude were alternated for the ten rows in the grid.
