# Supplementary material for: Early detection of human impacts using acoustic monitoring: An example with forest elephants
Source: PLoS One. 2024 Jul 26;19(7):e0306932. doi: 10.1371/journal.pone.0306932 (PMC11280225; doi:10.1371/journal.pone.0306932)
Supplement: S2 Text — (PDF) [file pone.0306932.s002.pdf]

S2 Text. Swift acoustic recorder detection distance for rumble calls. The Swift recorder uses an omnidirectional electret condenser style microphone (manufactured by PUI Audio Inc.®), that successfully records elephant rumble vocalizations with fundamental frequencies down to about 9 Hz. To calculate the distance at which an elephant rumble vocalization would be recorded by a Swift recorder deployed in Central African rainforest, we conducted an experiment at the Dzanga Bai in the Central African Republic. S2a Fig. shows the configuration of an acoustic array for localization and a transect of Swift recorders to estimate detection distance. Nine BAR-LT sound recorders (Frontier Labs, Australia) were deployed around the perimeter of the forest clearing. These use a GPS synchronized time stamp on sound files which allows for localization of identified signals registered by multiple BAR-LT units. Source locations were calculated using the correlation sum location estimation (CSE) algorithm (Kurt Fristrup and Kathryn Cortopassi, pers. comm., used in Mennill et al. 2006 (1)), implemented in the Extensible Bioacoustics Tool XBAT (2). Uncertainty in the location estimate was within 6 m, given the array geometry and a speed of sound estimated to be 350 m/s given average temperature and humidity. Because source location estimation is most accurate for calls originating within the perimeter of an acoustic array, we used the 1361 rumble calls originating in the central part of the clearing for analysis (Fig a). The line of Swift recorders was set to be roughly perpendicular to this part of the clearing, and so sound signals were propagating through the same part of forest to each of the Swift locations.

1. Mennill DJ, Burt JM, Fristrup KM, Vehrencamp SL. Accuracy of an acoustic location system for monitoring the position of duetting songbirds in tropical forest. Journal of the Acoustical Society of America. 2006;119:2832-9.

2. Figueroa H. Extensible Bioacoustics Tool (XBAT). 6.0.1 ed. Ithaca, NY: Cornell Bioacoustics Research Program; 2006.

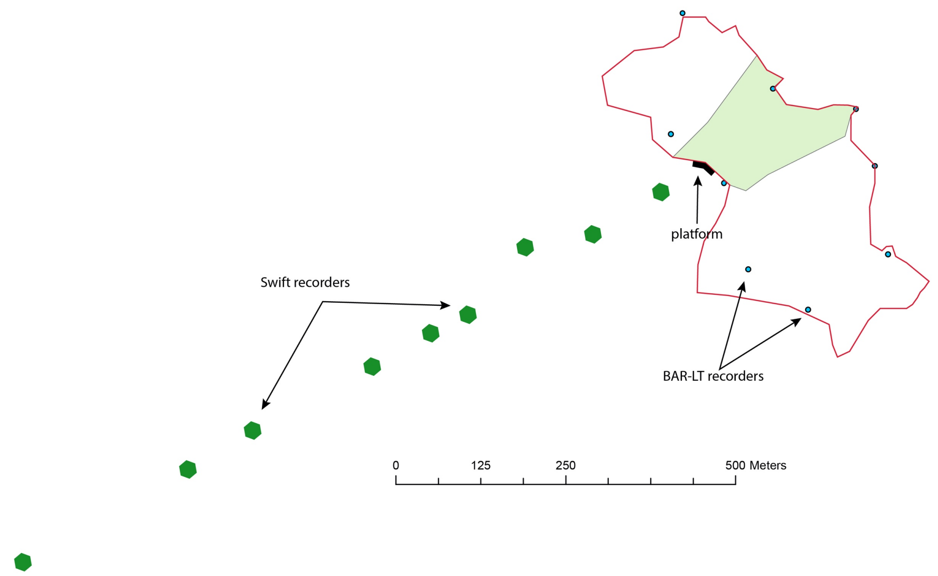

Fig a. Configuration of recording units used to localize the source of rumble calls (BAR-LT) and the transect of Swift recorders used to measure detection distance. Red line=perimeter of Dzanga Bai, green area=restricted source location of calls used in analysis.

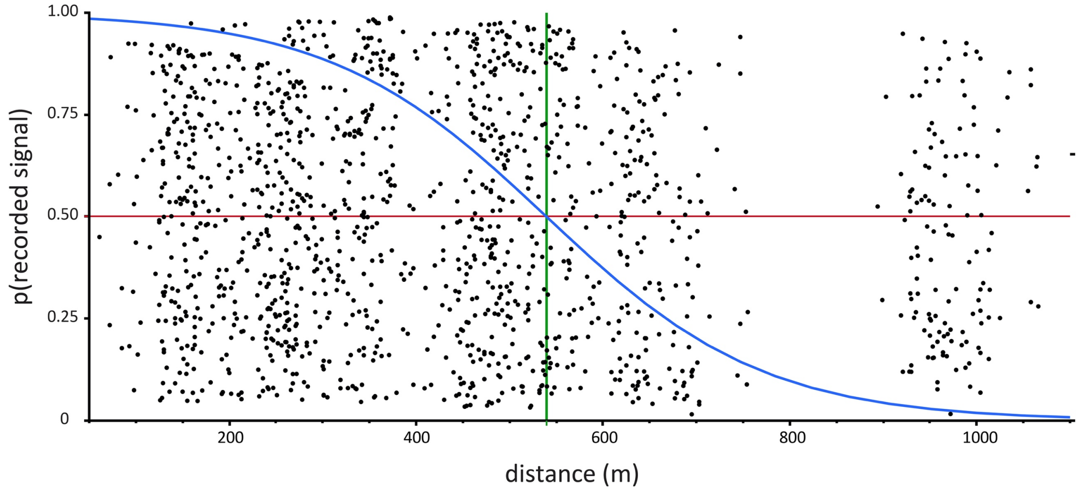

Fig b. Plot of logistic regression model used to estimate the detection distance of an elephant rumble vocalization, using Swift acoustic recorders. Shown is the logistic curve for the model, including only source locations in front of the observation platform (S2a Fig). GREEN vertical = 540m - the detection distance of an average rumble. Log odds of yes/no = 4.63 -.00858 (distance), n=1361 rumbles.
