# Supplementary material for: Early detection of human impacts using acoustic monitoring: An example with forest elephants
Source: PLoS One. 2024 Jul 26;19(7):e0306932. doi: 10.1371/journal.pone.0306932 (PMC11280225; doi:10.1371/journal.pone.0306932)
Supplement: S1 Table — Binomial model. (PDF) [file pone.0306932.s003.pdf]

S1 Table. **Parameter estimates for entire study system. Binomial model**.

**Parameter**			**DF**	**Estimate**	**SE**	**Wald CL**		**Wald ChiSq**	**P> ChiSq**
**Intercept**			1	-1.0799	0.1014	-1.2787	-0.8812	113.36	<.0001
**year**	**1**		1	0.9847	0.1017	0.7854	1.1839	93.82	<.0001
**year**	**2**		1	0.4145	0.1059	0.2070	0.6220	15.33	<.0001
**year**	**3**		1	0.6061	0.1105	0.3894	0.8227	30.07	<.0001
**year**	**4**		0	0.0000	0.0000	0.0000	0.0000	.	.
**stratum**	**inactive logging**		1	0.5764	0.0988	0.3828	0.7699	34.06	<.0001
**stratum**	**active logging**		1	0.2994	0.1562	-0.0068	0.6055	3.67	0.0553
**stratum**	**national park**		0	0.0000	0.0000	0.0000	0.0000	.	.
**season**	**dry**		1	0.0831	0.0953	-0.1037	0.2699	0.76	0.3833
**season**	**wet**		0	0.0000	0.0000	0.0000	0.0000	.	.
**call Density**			1	-0.0057	0.0003	-0.0064	-0.0051	282.32	<.0001
**forest**	**mono**		1	-0.6109	0.1808	-0.9652	-0.2567	11.42	0.0007
**forest**	**open**		1	2.1999	0.1099	1.9844	2.4153	400.50	<.0001
**forest**	**mixed**		0	0.0000	0.0000	0.0000	0.0000	.	.
**year*stratum**	**1**	**inactive logging**	1	-0.3384	0.1028	-0.5398	-0.1370	10.85	0.0010
**year*stratum**	**1**	**active logging**	1	-0.1506	0.1611	-0.4664	0.1651	0.87	0.3498
**year*stratum**	**1**	**national park**	0	0.0000	0.0000	0.0000	0.0000	.	.
**year*stratum**	**2**	**inactive logging**	1	-0.1533	0.1099	-0.3687	0.0621	1.95	0.1631
**year*stratum**	**2**	**active logging**	1	0.6726	0.1740	0.3316	1.0137	14.94	0.0001
**year*stratum**	**2**	**national park**	0	0.0000	0.0000	0.0000	0.0000	.	.
**year*stratum**	**3**	**inactive logging**	1	-0.6814	0.1144	-0.9056	-0.4572	35.49	<.0001
**year*stratum**	**3**	**active logging**	1	-0.1031	0.1775	-0.4510	0.2448	0.34	0.5614
**year*stratum**	**3**	**national park**	0	0.0000	0.0000	0.0000	0.0000	.	.
**year*stratum**	**4**	**inactive logging**	0	0.0000	0.0000	0.0000	0.0000	.	.

Parameter			DF	Estimate	SE	Wald CL		Wald ChiSq	P> ChiSq
**year*stratum**	**4**	**active logging**	0	0.0000	0.0000	0.0000	0.0000	.	.
**year*stratum**	**4**	**national park**	0	0.0000	0.0000	0.0000	0.0000	.	.
**year*season**	**1**	**dry**	1	-0.0695	0.0994	-0.2643	0.1254	0.49	0.4847
**year*season**	**1**	**wet**	0	0.0000	0.0000	0.0000	0.0000	.	.
**year*season**	**2**	**dry**	1	0.1008	0.1027	-0.1005	0.3021	0.96	0.3264
**year*season**	**2**	**wet**	0	0.0000	0.0000	0.0000	0.0000	.	.
**year*season**	**3**	**dry**	1	0.2072	0.1086	-0.0057	0.4201	3.64	0.0565
**year*season**	**3**	**wet**	0	0.0000	0.0000	0.0000	0.0000	.	.
**year*season**	**4**	**dry**	0	0.0000	0.0000	0.0000	0.0000	.	.
**year*season**	**4**	**wet**	0	0.0000	0.0000	0.0000	0.0000	.	.
**year*forest**	**1**	**mono**	1	0.2446	0.1833	-0.1146	0.6039	1.78	0.1820
**year*forest**	**1**	**open**	1	-0.7292	0.1095	-0.9439	-0.5146	44.34	<.0001
**year*forest**	**1**	**mixed**	0	0.0000	0.0000	0.0000	0.0000	.	.
**year*forest**	**2**	**mono**	1	0.3787	0.1944	-0.0023	0.7597	3.80	0.0514
**year*forest**	**2**	**open**	1	-0.6972	0.1147	-0.9221	-0.4724	36.94	<.0001
**year*forest**	**2**	**mixed**	0	0.0000	0.0000	0.0000	0.0000	.	.
**year*forest**	**3**	**mono**	1	0.2319	0.2026	-0.1651	0.6289	1.31	0.2522
**year*forest**	**3**	**open**	1	-0.5897	0.1187	-0.8222	-0.3571	24.69	<.0001
**year*forest**	**3**	**mixed**	0	0.0000	0.0000	0.0000	0.0000	.	.
**year*forest**	**4**	**mono**	0	0.0000	0.0000	0.0000	0.0000	.	.
**year*forest**	**4**	**open**	0	0.0000	0.0000	0.0000	0.0000	.	.
**year*forest**	**4**	**mixed**	0	0.0000	0.0000	0.0000	0.0000	.	.
**season*forest**	**dry**	**mono**	1	-0.3765	0.0834	-0.5399	-0.2131	20.39	<.0001
**season*forest**	**dry**	**open**	1	-0.1592	0.0688	-0.2941	-0.0243	5.35	0.0208
**season*forest**	**dry**	**mixed**	0	0.0000	0.0000	0.0000	0.0000	.	.
**season*forest**	**wet**	**mono**	0	0.0000	0.0000	0.0000	0.0000	.	.
**season*forest**	**wet**	**open**	0	0.0000	0.0000	0.0000	0.0000	.	.
**season*forest**	**wet**	**mixed**	0	0.0000	0.0000	0.0000	0.0000	.	.
**call Density*season**	**dry**		1	0.0028	0.0003	0.0021	0.0035	65.32	<.0001
**call Density*season**	**wet**		0	0.0000	0.0000	0.0000	0.0000	.	.
**call Density*forest**	**mono**		1	0.0049	0.0009	0.0032	0.0066	30.54	<.0001
**call Density*forest**	**open**		1	0.0027	0.0004	0.0019	0.0034	44.26	<.0001

Parameter		DF	Estimate	SE	Wald CL		Wald ChiSq	P> ChiSq
**call Density*forest**	**mixed**	0	0.0000	0.0000	0.0000	0.0000	.	.
**Scale**		0	1.0000	0.0000	1.0000	1.0000		
