# Supplementary material for: Early detection of human impacts using acoustic monitoring: An example with forest elephants
Source: PLoS One. 2024 Jul 26;19(7):e0306932. doi: 10.1371/journal.pone.0306932 (PMC11280225; doi:10.1371/journal.pone.0306932)
Supplement: S2 Table — Basic binomial model. (PDF) [file pone.0306932.s004.pdf]

S2 Table. **Parameter estimates for the national park stratum. Basic binomial model.**

**Parameter**			**DF**	**Estimate**	**SE**	**Wald CL**		**Wald ChiSq**	**Pr > ChiSq**
**Intercept**			1	-0.0505	0.1383	-0.3216	0.2206	0.13	0.7150
**year**	**1**		1	-0.0722	0.1391	-0.3448	0.2004	0.27	0.6037
**year**	**2**		1	-0.7154	0.1433	-0.9963	-0.4346	24.93	<.0001
**year**	**3**		1	-0.7425	0.1505	-1.0375	-0.4475	24.34	<.0001
**year**	**4**		0	0.0000	0.0000	0.0000	0.0000	.	.
**season**	**dry**		1	-0.9752	0.1525	-1.2741	-0.6763	40.89	<.0001
**season**	**wet**		0	0.0000	0.0000	0.0000	0.0000	.	.
**forest**	**mono**		1	-1.2867	0.2659	-1.8078	-0.7656	23.42	<.0001
**forest**	**open**		1	0.9312	0.1602	0.6172	1.2452	33.78	<.0001
**forest**	**mixed**		0	0.0000	0.0000	0.0000	0.0000	.	.
**call Density**			1	-0.0051	0.0004	-0.0059	-0.0042	132.00	<.0001
**year*season**	**1**	**dry**	1	0.7905	0.1504	0.4958	1.0852	27.64	<.0001
**year*season**	**1**	**wet**	0	0.0000	0.0000	0.0000	0.0000	.	.
**year*season**	**2**	**dry**	1	0.9515	0.1616	0.6347	1.2682	34.66	<.0001
**year*season**	**2**	**wet**	0	0.0000	0.0000	0.0000	0.0000	.	.
**year*season**	**3**	**dry**	1	1.2592	0.1746	0.9171	1.6014	52.02	<.0001
**year*season**	**3**	**wet**	0	0.0000	0.0000	0.0000	0.0000	.	.
**year*season**	**4**	**dry**	0	0.0000	0.0000	0.0000	0.0000	.	.
**year*season**	**4**	**wet**	0	0.0000	0.0000	0.0000	0.0000	.	.
**year*forest**	**1**	**mono**	1	0.7643	0.2659	0.2431	1.2854	8.26	0.0040
**year*forest**	**1**	**open**	1	0.2505	0.1518	-0.0471	0.5480	2.72	0.0990
**year*forest**	**1**	**mixed**	0	0.0000	0.0000	0.0000	0.0000	.	.
**year*forest**	**2**	**mono**	1	0.9756	0.2853	0.4163	1.5349	11.69	0.0006
**year*forest**	**2**	**open**	1	1.1762	0.1919	0.8001	1.5524	37.56	<.0001
**year*forest**	**2**	**mixed**	0	0.0000	0.0000	0.0000	0.0000	.	.
**year*forest**	**3**	**mono**	1	1.6029	0.2986	1.0175	2.1882	28.81	<.0001
**year*forest**	**3**	**open**	1	1.1657	0.1743	0.8241	1.5074	44.73	<.0001
**year*forest**	**3**	**mixed**	0	0.0000	0.0000	0.0000	0.0000	.	.
**year*forest**	**4**	**mono**	0	0.0000	0.0000	0.0000	0.0000	.	.
**year*forest**	**4**	**open**	0	0.0000	0.0000	0.0000	0.0000	.	.
**year*forest**	**4**	**mixed**	0	0.0000	0.0000	0.0000	0.0000	.	.
**season*forest**	**dry**	**mono**	1	-0.8483	0.1396	-1.1219	-0.5747	36.93	<.0001

Parameter			DF	Estimate	SE	Wald CL		Wald ChiSq	Pr > ChiSq
**season*forest**	**dry**	**open**	1	-0.7669	0.1103	-0.9831	-0.5508	48.36	<.0001
**season*forest**	**dry**	**mixed**	0	0.0000	0.0000	0.0000	0.0000	.	.
**season*forest**	**wet**	**mono**	0	0.0000	0.0000	0.0000	0.0000	.	.
**season*forest**	**wet**	**open**	0	0.0000	0.0000	0.0000	0.0000	.	.
**season*forest**	**wet**	**mixed**	0	0.0000	0.0000	0.0000	0.0000	.	.
**call Density*season**	**dry**		1	0.0130	0.0010	0.0111	0.0149	185.50	<.0001
**call Density*season**	**wet**		0	0.0000	0.0000	0.0000	0.0000	.	.
**call Density*forest**	**mono**		1	0.0051	0.0013	0.0027	0.0076	16.63	<.0001
**call Density*forest**	**open**		1	0.0054	0.0009	0.0037	0.0071	39.72	<.0001
**call Density*forest**	**mixed**		0	0.0000	0.0000	0.0000	0.0000	.	.
**Scale**			0	1.0000	0.0000	1.0000	1.0000		
