# Supplementary material for: Early detection of human impacts using acoustic monitoring: An example with forest elephants
Source: PLoS One. 2024 Jul 26;19(7):e0306932. doi: 10.1371/journal.pone.0306932 (PMC11280225; doi:10.1371/journal.pone.0306932)
Supplement: S3 Table — Basic binomial model. (PDF) [file pone.0306932.s005.pdf]

S3 Table. **Parameter estimates for the inactive logging stratum. Basic binomial model.**

Parameter			DF	Estimate	SE	Wald CL		Wald ChiSq	Pr > ChiSq
Intercept			1	-0.8710	0.1280	-1.1220	-0.6201	46.28	<.0001
year	1		1	1.2415	0.1267	0.9931	1.4899	95.97	<.0001
year	2		1	1.0535	0.1321	0.7946	1.3124	63.61	<.0001
year	3		1	0.6290	0.1429	0.3490	0.9091	19.38	<.0001
year	4		0	0.0000	0.0000	0.0000	0.0000	.	.
season	dry		1	0.3500	0.1775	0.0021	0.6980	3.89	0.0486
season	wet		0	0.0000	0.0000	0.0000	0.0000	.	.
forest	open		1	3.3448	0.2025	2.9479	3.7418	272.78	<.0001
forest	mixed		0	0.0000	0.0000	0.0000	0.0000	.	.
call Density			1	-0.0103	0.0006	-0.0115	-0.0091	288.54	<.0001
year*season	1	dry	1	-0.3725	0.1890	-0.7430	-0.0020	3.88	0.0488
year*season	1	wet	0	0.0000	0.0000	0.0000	0.0000	.	.
year*season	2	dry	1	-0.3454	0.1898	-0.7175	0.0266	3.31	0.0688
year*season	2	wet	0	0.0000	0.0000	0.0000	0.0000	.	.
year*season	3	dry	1	0.1658	0.1979	-0.2221	0.5537	0.70	0.4022
year*season	3	wet	0	0.0000	0.0000	0.0000	0.0000	.	.
year*season	4	dry	0	0.0000	0.0000	0.0000	0.0000	.	.
year*season	4	wet	0	0.0000	0.0000	0.0000	0.0000	.	.
year*forest	1	open	1	-2.1248	0.2006	-2.5180	-1.7315	112.15	<.0001
year*forest	1	mixed	0	0.0000	0.0000	0.0000	0.0000	.	.
year*forest	2	open	1	-2.4864	0.2032	-2.8847	-2.0881	149.68	<.0001
year*forest	2	mixed	0	0.0000	0.0000	0.0000	0.0000	.	.
year*forest	3	open	1	-2.4542	0.2093	-2.8645	-2.0439	137.43	<.0001
year*forest	3	mixed	0	0.0000	0.0000	0.0000	0.0000	.	.
year*forest	4	open	0	0.0000	0.0000	0.0000	0.0000	.	.
year*forest	4	mixed	0	0.0000	0.0000	0.0000	0.0000	.	.
season*forest	dry	open	1	0.3889	0.1014	0.1902	0.5877	14.71	0.0001
season*forest	dry	mixed	0	0.0000	0.0000	0.0000	0.0000	.	.
season*forest	wet	open	0	0.0000	0.0000	0.0000	0.0000	.	.
season*forest	wet	mixed	0	0.0000	0.0000	0.0000	0.0000	.	.
call Density*season	dry		1	-0.0006	0.0004	-0.0014	0.0002	2.20	0.1380
call Density*season	wet		0	0.0000	0.0000	0.0000	0.0000	.	.

Parameter		DF	Estimate	SE	Wald CL		Wald ChiSq	Pr > ChiSq
call Density*forest	open	1	0.0079	0.0007	0.0066	0.0092	146.19	<.0001
call Density*forest	mixed	0	0.0000	0.0000	0.0000	0.0000	.	.
Scale		0	1.0000	0.0000	1.0000	1.0000		
