# Supplementary material for: Early detection of human impacts using acoustic monitoring: An example with forest elephants
Source: PLoS One. 2024 Jul 26;19(7):e0306932. doi: 10.1371/journal.pone.0306932 (PMC11280225; doi:10.1371/journal.pone.0306932)
Supplement: S4 Table — Modified binomial model. (PDF) [file pone.0306932.s006.pdf]

S4 Table. **Parameter estimates for the active logging stratum. Binomial model is modified from the basic model for other strata in order to include logging exposure parameters and interactions.**

Parameter			DF	Estimate	SE	Wald CL		Wald ChiSq	Pr > ChiSq
Intercept			1	-0.2627	0.3463	-0.9415	0.4161	0.58	0.4481
year	1		1	0.6261	0.2720	0.0930	1.1593	5.30	0.0213
year	2		1	1.0233	0.2901	0.4547	1.5919	12.44	0.0004
year	3		1	1.0170	0.2790	0.4702	1.5637	13.29	0.0003
year	4		0	0.0000	0.0000	0.0000	0.0000	.	.
season	dry		1	-0.4197	0.7602	-1.9098	1.0704	0.30	0.5809
season	wet		0	0.0000	0.0000	0.0000	0.0000	.	.
forest	mono		1	20.5729	0.2646	20.0544	21.0914	6047.37	<.0001
forest	mixed		0	0.0000	0.0000	0.0000	0.0000	.	.
exposure	active		1	-0.5053	0.2386	-0.9728	-0.0377	4.49	0.0342
exposure	done1		1	0.0976	0.3039	-0.4980	0.6931	0.10	0.7481
exposure	done2		1	-0.6907	0.2912	-1.2614	-0.1200	5.63	0.0177
exposure	done3		1	-1.0355	0.2603	-1.5456	-0.5254	15.83	<.0001
exposure	done4		1	-1.2732	0.2645	-1.7916	-0.7547	23.17	<.0001
exposure	done5		1	-1.5493	0.3215	-2.1796	-0.9191	23.22	<.0001
exposure	done6		1	-20.3653	0.7642	-21.8632	-18.8675	710.11	<.0001
exposure	preExp		0	0.0000	0.0000	0.0000	0.0000	.	.
call Density			1	-0.0705	0.0150	-0.1000	-0.0411	22.06	<.0001
year*season	1	dry	1	1.5142	0.6029	0.3325	2.6959	6.31	0.0120
year*season	1	wet	0	0.0000	0.0000	0.0000	0.0000	.	.
year*season	2	dry	1	1.6025	0.6000	0.4266	2.7785	7.13	0.0076
year*season	2	wet	0	0.0000	0.0000	0.0000	0.0000	.	.
year*season	3	dry	1	0.7654	0.5453	-0.3033	1.8342	1.97	0.1604
year*season	3	wet	0	0.0000	0.0000	0.0000	0.0000	.	.
year*season	4	dry	0	0.0000	0.0000	0.0000	0.0000	.	.
year*season	4	wet	0	0.0000	0.0000	0.0000	0.0000	.	.
year*forest	1	mono	1	-20.1678	0.3004	-20.7566	-19.5790	4506.97	<.0001
year*forest	1	mixed	0	0.0000	0.0000	0.0000	0.0000	.	.
year*forest	2	mono	1	-20.1596	0.3093	-20.7659	-19.5532	4246.81	<.0001
year*forest	2	mixed	0	0.0000	0.0000	0.0000	0.0000	.	.
year*forest	3	mono	0	-19.4395	0.0000	-19.4395	-19.4395	.	.

Parameter			DF	Estimate	SE	Wald CL		Wald ChiSq	Pr > ChiSq
**year*forest**	**3**	**mixed**	0	0.0000	0.0000	0.0000	0.0000	.	.
**year*forest**	**4**	**mono**	0	0.0000	0.0000	0.0000	0.0000	.	.
**year*forest**	**4**	**mixed**	0	0.0000	0.0000	0.0000	0.0000	.	.
**season*forest**	**dry**	**mono**	1	0.5385	0.2331	0.0816	0.9955	5.34	0.0209
**season*forest**	**dry**	**mixed**	0	0.0000	0.0000	0.0000	0.0000	.	.
**season*forest**	**wet**	**mono**	0	0.0000	0.0000	0.0000	0.0000	.	.
**season*forest**	**wet**	**mixed**	0	0.0000	0.0000	0.0000	0.0000	.	.
**season*exposure**	**dry**	**active**	1	-2.6551	0.5039	-3.6428	-1.6674	27.76	<.0001
**season*exposure**	**dry**	**done1**	1	-0.7795	0.5902	-1.9363	0.3773	1.74	0.1866
**season*exposure**	**dry**	**done2**	1	0.1694	0.6120	-1.0302	1.3689	0.08	0.7820
**season*exposure**	**dry**	**done3**	1	-2.3405	0.5218	-3.3632	-1.3178	20.12	<.0001
**season*exposure**	**dry**	**done4**	1	-1.3246	0.5239	-2.3516	-0.2977	6.39	0.0115
**season*exposure**	**dry**	**done5**	1	-2.3770	0.6602	-3.6710	-1.0831	12.96	0.0003
**season*exposure**	**dry**	**done6**	1	-2.2609	1.3013	-4.8114	0.2896	3.02	0.0823
**season*exposure**	**dry**	**preExp**	0	0.0000	0.0000	0.0000	0.0000	.	.
**season*exposure**	**wet**	**active**	0	0.0000	0.0000	0.0000	0.0000	.	.
**season*exposure**	**wet**	**done1**	0	0.0000	0.0000	0.0000	0.0000	.	.
**season*exposure**	**wet**	**done2**	0	0.0000	0.0000	0.0000	0.0000	.	.
**season*exposure**	**wet**	**done3**	0	0.0000	0.0000	0.0000	0.0000	.	.
**season*exposure**	**wet**	**done4**	0	0.0000	0.0000	0.0000	0.0000	.	.
**season*exposure**	**wet**	**done5**	0	0.0000	0.0000	0.0000	0.0000	.	.
**season*exposure**	**wet**	**done6**	0	0.0000	0.0000	0.0000	0.0000	.	.
**season*exposure**	**wet**	**preExp**	0	0.0000	0.0000	0.0000	0.0000	.	.
**call Density*season**	**dry**		1	0.0103	0.0031	0.0043	0.0163	11.36	0.0008
**call Density*season**	**wet**		0	0.0000	0.0000	0.0000	0.0000	.	.
**call Density*forest**	**mono**		1	-0.0058	0.0040	-0.0137	0.0020	2.11	0.1461
**call Density*forest**	**mixed**		0	0.0000	0.0000	0.0000	0.0000	.	.
**call Density*exposure**	**active**		1	0.0945	0.0154	0.0642	0.1247	37.44	<.0001
**call Density*exposure**	**done1**		1	0.0234	0.0172	-0.0104	0.0572	1.85	0.1743
**call Density*exposure**	**done2**		1	0.0630	0.0156	0.0323	0.0937	16.22	<.0001
**call Density*exposure**	**done3**		1	0.0778	0.0157	0.0472	0.1085	24.73	<.0001
**call Density*exposure**	**done4**		1	0.0838	0.0155	0.0534	0.1142	29.22	<.0001
**call Density*exposure**	**done5**		1	0.0645	0.0157	0.0336	0.0953	16.76	<.0001

Parameter		DF	Estimate	SE	Wald CL		Wald ChiSq	Pr > ChiSq
**call Density*exposure**	**done6**	1	0.0516	0.0445	-0.0356	0.1388	1.35	0.2457
**call Density*exposure**	**preExp**	0	0.0000	0.0000	0.0000	0.0000	.	.
**Scale**		0	1.0000	0.0000	1.0000	1.0000		
