# Supplementary material for: Early detection of human impacts using acoustic monitoring: An example with forest elephants
Source: PLoS One. 2024 Jul 26;19(7):e0306932. doi: 10.1371/journal.pone.0306932 (PMC11280225; doi:10.1371/journal.pone.0306932)
Supplement: S5 Table — Zero-inflated binomial model of call density. (PDF) [file pone.0306932.s007.pdf]

S5 Table. **Parameter estimates for the active logging stratum. Zero-inflated binomial model of call density**.

Parameter			DF	Estimate	SE	Wald CL		Wald ChiSq	Pr > ChiSq
Intercept			1	1.8584	0.3177	1.2358	2.4810	34.23	<.0001
year	1		1	-0.6926	0.2819	-1.2452	-0.1401	6.04	0.0140
year	2		1	-0.7050	0.2909	-1.2751	-0.1349	5.87	0.0154
year	3		1	-0.4947	0.2725	-1.0289	0.0394	3.30	0.0695
year	4		0	0.0000	0.0000	0.0000	0.0000	.	.
season	dry		1	0.0038	0.1206	-0.2326	0.2402	0.00	0.9746
season	wet		0	0.0000	0.0000	0.0000	0.0000	.	.
forest	mono		1	0.2261	0.1420	-0.0522	0.5044	2.53	0.1114
forest	mixed		0	0.0000	0.0000	0.0000	0.0000	.	.
exposure	active		1	0.4593	0.1752	0.1159	0.8026	6.87	0.0087
exposure	done1		1	0.5753	0.2295	0.1254	1.0252	6.28	0.0122
exposure	done2		1	0.5061	0.2292	0.0567	0.9554	4.87	0.0273
exposure	done3		1	0.6469	0.1989	0.2570	1.0367	10.58	0.0011
exposure	done4		1	1.1170	0.2131	0.6993	1.5346	27.48	<.0001
exposure	done5		1	0.9613	0.2714	0.4294	1.4932	12.55	0.0004
exposure	done6		1	0.4867	0.3752	-0.2486	1.2220	1.68	0.1945
exposure	preExp		0	0.0000	0.0000	0.0000	0.0000	.	.
season*forest	dry	mono	1	0.6632	0.2403	0.1924	1.1341	7.62	0.0058
season*forest	dry	mixed	0	0.0000	0.0000	0.0000	0.0000	.	.
season*forest	wet	mono	0	0.0000	0.0000	0.0000	0.0000	.	.
season*forest	wet	mixed	0	0.0000	0.0000	0.0000	0.0000	.	.
Dispersion			1	1.1006	0.1096	0.9055	1.3378		
