# Supplementary material for: Early detection of human impacts using acoustic monitoring: An example with forest elephants
Source: PLoS One. 2024 Jul 26;19(7):e0306932. doi: 10.1371/journal.pone.0306932 (PMC11280225; doi:10.1371/journal.pone.0306932)
Supplement: S6 Table — (PDF) [file pone.0306932.s008.pdf]

S6 Table. **Forest type determination based on proportion of pixels in each of three classes (from Swider, 2023).**

site	%mixed	%monodominant	%open	forest type	stratum
nn01a	0.73	0.26	0.01	mixed	national park
nn01b	0.69	0.27	0.04	mixed	national park
nn01c	0.92	0.04	0.03	mixed	national park
nn01d	0.71	0.29	0.01	mixed	national park
nn01e	0.68	0.20	0.12	mixed	national park
nn01f	0.35	0.64	0.01	mono	national park
nn01g	0.67	0.26	0.07	mixed	national park
nn02a	0.55	0.44	0.00	mixed	national park
nn02b	0.63	0.26	0.11	mixed	national park
nn02c	0.47	0.43	0.09	mixed	national park
nn02d	0.77	0.22	0.01	mixed	national park
nn02e	0.70	0.30	0.00	mixed	national park
nn02f	0.48	0.51	0.00	mono	national park
nn02g	0.88	0.08	0.05	mixed	national park
nn03a	0.91	0.03	0.06	mixed	national park
nn03b	0.36	0.63	0.01	mono	national park
nn03c	0.24	0.55	0.20	mono	national park
nn03d	0.93	0.06	0.01	mixed	national park
nn03e	0.41	0.42	0.16	mono	national park
nn03f	0.34	0.61	0.05	mono	national park
nn03g	0.71	0.26	0.02	mixed	national park
nn04a	0.87	0.07	0.06	mixed	national park
nn04b	0.59	0.31	0.10	mixed	national park
nn04c	0.87	0.11	0.02	mixed	national park
nn04d	0.46	0.30	0.25	mixed	active logging
nn04e	0.83	0.14	0.03	mixed	active logging
nn04f	0.71	0.27	0.02	mixed	active logging
nn05a	0.86	0.10	0.03	mixed	national park
nn05b	0.63	0.36	0.02	mixed	national park
nn05c	0.84	0.11	0.05	mixed	active logging
nn05d	0.86	0.10	0.04	mixed	active logging
nn05e	0.75	0.18	0.07	mixed	active logging
nn05f	0.77	0.21	0.01	mixed	active logging
nn06a	0.15	0.35	0.50	open	inactive logging
nn06b	0.42	0.07	0.51	open	national park
nn06c	0.54	0.28	0.18	mixed	national park
nn06d	0.45	0.53	0.02	mono	active logging
nn06e	0.38	0.61	0.01	mono	active logging

nn06f	0.62	0.36	0.02	mixed	active logging
nn07a	0.84	0.06	0.10	mixed	inactive logging
nn07b	0.84	0.06	0.09	mixed	inactive logging
nn07c	0.15	0.38	0.47	open	national park
nn08a	0.73	0.22	0.05	mixed	inactive logging
nn08b	0.80	0.09	0.11	mixed	inactive logging
nn08c	0.34	0.02	0.65	open	inactive logging
nn09a	0.53	0.44	0.03	mixed	inactive logging
nn09b	0.89	0.07	0.04	mixed	inactive logging
nn09c	0.88	0.08	0.04	mixed	inactive logging
nn10a	0.86	0.07	0.07	mixed	inactive logging
nn10b	0.58	0.39	0.03	mixed	inactive logging
